# Machine learning assisted nanobeam X-ray diffraction based analysis on hydride vapor-phase epitaxy GaN

**DOI:** 10.1107/S1600576725004169

**Published:** 2025-07-08

**Authors:** Zhendong Wu, Yusuke Hayashi, Tetsuya Tohei, Kazushi Sumitani, Yasuhiko Imai, Shigeru Kimura, Akira Sakai

**Affiliations:** a Graduate School of Engineering Science Osaka University 1-3 Machikaneyama-cho, Toyonaka Osaka 560-8531 Japan; b National Institute for Materials Science 1-2-1 Sengen Tsukuba Ibaraki305-0047 Japan; c Japan Synchrotron Radiation Research Institute 1-1-1 Kouto, Sayo-cho Sayo Hyôgo 679-5198 Japan; Ecole National Supérieure des Mines, Saint-Etienne, France

**Keywords:** hydride vapor-phase epitaxy films, HVPE GaN wafers, machine learning, X-ray diffraction, nanoXRD, crystal growth

## Abstract

Nanobeam X-ray diffraction (nanoXRD) is a powerful tool for collecting *in situ* crystal structure information with high spatial resolution and data acquisition rate. However, analyzing the enormous amount of data produced by these high-throughput experiments for defect recognition or discovering hidden structural features becomes challenging. Machine learning (ML) methods have become attractive recently due to their outstanding performance in analyzing large data sets. This research utilizes an ML algorithm, uniform manifold approximation and projection (UMAP), to enhance the nanoXRD-based crystal structure analysis of a cross-sectional hydride vapor-phase epitaxy GaN wafer. Compared with the results obtained by conventional fitting, UMAP gives a more precise categorization of crystal structure based on the raw three-dimensional ω–2θ–φ diffraction patterns. The property that UMAP embeds the high-dimensional data while retaining the data structure is valuable in guiding the analysis of nanoXRD profiles. This research also demonstrates the capability of UMAP in analyzing other spectroscopic or diffraction data sets to guide crystal structure investigations.

## Abstract 2

Using a machine learning method, this study enhances crystal structure analysis for a cross-sectional hydride vapor-phase epitaxy GaN wafer, revealing hidden features and aiding structural investigations, and outperforming the conventional method.

## 1. Introduction

X-ray diffraction (XRD) is a fundamental technology in structure and phase identification and materials discovery, and it is diversely applied in research on batteries, catalysts, semiconductors *etc.* (Ahmad *et al.*, 2021 ▸; Billinge *et al.*, 2019 ▸; Hua *et al.*, 2021 ▸; Kusne *et al.*, 2014 ▸; Shadike *et al.*, 2021 ▸). Development in measurement automation, detectors and X-ray sources has dramatically increased the resolution and acquisition efficiency of diffraction patterns. In recent years, the highly collimated and monochromatic photon flux produced in synchrotron radiation facilities has given rise to the nanobeam XRD (nanoXRD) technique, which can reach a high spatial resolution on a nanometre scale. Synchrotron X-ray sources and automation systems ensure high-throughput nanoXRD experiments, including *in situ* measurements with high spatial and time resolution. Meanwhile, it has been reported that, thanks to the development of two-dimensional (2D) detectors, nanoXRD can provide diffraction data in three-dimensional (3D) reciprocal space to help us obtain position- and time-dependent crystalline information (Aoyagi *et al.*, 2022 ▸; Hamachi *et al.*, 2024 ▸; Imai *et al.*, 2019 ▸; Kamada *et al.*, 2016 ▸; Nagaoka *et al.*, 2023 ▸; Onabe *et al.*, 2024 ▸; Shida *et al.*, 2017 ▸; Shida *et al.*, 2018 ▸; Shida *et al.*, 2019 ▸; Shiomi *et al.*, 2021 ▸).

However, challenges come with the enormous number of diffraction patterns produced by nanoXRD measurements. Outstanding advances in data acquisition techniques have outpaced data analysis methods, which remain time consuming and demand experienced researchers. One of the important applications of nanoXRD is the analysis of localized structures and mapping of these structures using position-dependent measurements for single-crystal materials, such as semiconductor epitaxial films and bulk crystals (Hamachi *et al.*, 2024 ▸; Kamada *et al.*, 2016 ▸; Onabe *et al.*, 2024 ▸; Shida *et al.*, 2017 ▸; Shida *et al.*, 2018 ▸; Shida *et al.*, 2019 ▸). The diffraction patterns from a single crystal contain local crystallinity information, including strain and defects induced by various sources during crystal growth. To investigate these crystal structures in more detail, we have to refine and extract the data of interest from raw diffraction images. Conventionally, a refinement process includes (i) integration, transforming raw images into 1D diffraction intensity spectra, and (ii) subsequent curve fitting to extract the peak or full width at half-maximum (FWHM) from the XRD spectra obtained at all measurement positions for mapping, *e.g.* diffraction angle 2θ values for lattice spacing and the incident angle ω for lattice plane tilting.

The integration process is essential in the conventional refinement process, since we lack a technique for efficiently and directly analyzing the thousands of raw 3D diffraction patterns in ω–2θ–φ space (φ is the angle of lattice plane twisting). So we have to integrate the intensity along the other angular axes to transform the raw 3D data into a 1D ω, 2θ or φ intensity spectrum. A complete crystal structure analysis usually needs thousands of sampling points from the sample, *i.e.* 2D or 3D sampling in real space or 4D in temporal–spatial sampling, and each point can have 3D data in reciprocal space. The vast amount of raw 3D diffraction data makes the conventional analysis process of extraction of hidden structural information extremely challenging.

The information obtained from the refinement of position-dependent nanoXRD diffraction patterns helps us investigate local crystal structure changes. However, with the conventional method of data analysis we often encounter some critical problems related to the refinement process described in the previous section. The first crucial point is that, when the raw 3D data are integrated into 1D data for further analysis, some useful and valuable information relating to the crystal structure may be lost during the integration process. Another point is that we conventionally use Gaussian functions to fit the diffraction profiles. However, the fit is not certain when the diffraction profiles do not have a symmetric shape, especially when the crystallinity is degraded. It is essential to use the experience and expertise of scientists to address the various challenges encountered during data analysis.

Given that collecting and analyzing crystal information from the enormous amount of data by relying solely on human power is error prone, machine learning (ML) or deep learning (DL) can assist in classifying XRD patterns (Banko *et al.*, 2021 ▸; Chan *et al.*, 2021 ▸; Maffettone *et al.*, 2021*a* ▸; Maffettone *et al.*, 2021*b* ▸; Park *et al.*, 2017 ▸; Stanev *et al.*, 2018 ▸; Szymanski *et al.*, 2023 ▸). For most investigations that have applied ML or DL models to materials science, the researchers have focused on phase identification of mixtures of materials or organic samples based on the XRD spectra (Banko *et al.*, 2021 ▸; Chan *et al.*, 2021 ▸; Maffettone *et al.*, 2021*a* ▸; Maffettone *et al.*, 2021*b* ▸; Park *et al.*, 2017 ▸; Stanev *et al.*, 2018 ▸; Szymanski *et al.*, 2023 ▸). It has been reported that ML or DL approaches achieve expert accuracy in phase predictions and are robust against pattern perturbation caused by factors like texture, strain and mix­tures (Banko *et al.*, 2021 ▸; Maffettone *et al.*, 2021*a* ▸; Maffet­tone *et al.*, 2021*b* ▸; Park *et al.*, 2017 ▸; Stanev *et al.*, 2018 ▸). However, most previously reported ML or DL models are trained with known phase or component information on the mixture, *i.e.* simulated diffraction spectra based on preexisting physical knowledge are used to prepare the training data set. In con­trast, in bulk and epitaxial crystal structure analysis, the lack of a solid physical model that strictly describes the form­ation of defects or micro­structures during practical bulk crystal growth and the resulting XRD patterns prevents us from training an ML or DL model with simulation or known information.

To overcome these addressed challenges in the preparation of training data sets, we applied an unsupervised ML algorithm, uniform manifold approximation and projection (UMAP) (McInnes *et al.*, 2018*a* ▸; McInnes *et al.*, 2018*b* ▸), to analyze raw diffraction images directly. UMAP is a powerful dimensionality reduction algorithm that provides meaningful low-dimensional representations of complex data sets. Unlike classical approaches like principal component analysis (PCA) or multidimensional scaling (MDS), which rely on linear transformations, UMAP is designed to reveal nonlinear relationships (Roweis & Saul, 2000 ▸; Tenenbaum *et al.*, 2000 ▸) and is rooted in manifold learning and graph-based topology, making it well suited to complex high-dimensional data structures. In many real-world applications, data are collected in a high-dimensional space, while meaningful structures within the data often lie on a lower-dimensional manifold embedded in the high-dimensional space. Manifold learning, a subfield of machine learning, aims to uncover and represent lower-dimensional structures while preserving the geometric relationships in the data (Meilă & Zhang, 2023 ▸; Izenman, 2012 ▸). A conventionally known and popular manifold learning method is *t*-distributed stochastic neighborhood embedding (*t*-SNE) (Amir *et al.*, 2013 ▸; Van Der Maaten & Hinton, 2008 ▸). On the other hand, UMAP is widely reported to have been applied in biological research on single-cell data sets for dimensionality reduction, cluster identification and trajectory analysis with high efficiency (Becht *et al.*, 2019 ▸; Luo *et al.*, 2022 ▸; Sainburg *et al.*, 2021 ▸). Unlike the variational autoencoder (VAE), which is another popular artificial neural network algorithm that compresses input data into a low-dimensional latent space (Amarbayasgalan *et al.*, 2018 ▸; Banko *et al.*, 2021 ▸; Stein *et al.*, 2019 ▸), UMAP preserves the intrinsic geometric relationships within a data set without enforcing a generative model, whereas VAE learns a latent space by optimizing a probabilistic model to capture the data distribution for reconstruction. Such features of UMAP may find their merit in structure analysis based on vast diffraction data sets.

This work aims to address the challenges of preparing training data sets and to develop an ML-based method to facilitate crystal structure analysis. The crystal structure and defects in a bulk crystal are investigated using ML applied to a hydride vapor-phase epitaxy (HVPE) gallium nitride (GaN) wafer (Hamachi *et al.*, 2021 ▸; Hamachi *et al.*, 2023 ▸; Sato *et al.*, 2023 ▸), which serves as a test sample for collecting various diffraction data sets to evaluate the performance of the ML method. A series of nanoXRD measurements are performed to uncover the growth structure and defect formation processes through cross-sectional position-dependent nanoXRD analysis, producing high-dimensional raw diffraction data; 2D sampling in real space plus 3D sampling in reciprocal space results in a 5D hypercube of diffraction data. Our results demonstrate that UMAP has outstanding performance in clustering raw diffraction images, enabling the classification of crystal structures with less information loss than conventional methods. Moreover, UMAP effectively highlights regions of the sample that contain valuable information, guiding researchers towards deeper investigation.

## 2. Results

### 2.1. Details of nanoXRD measurements

**Figure 1:**
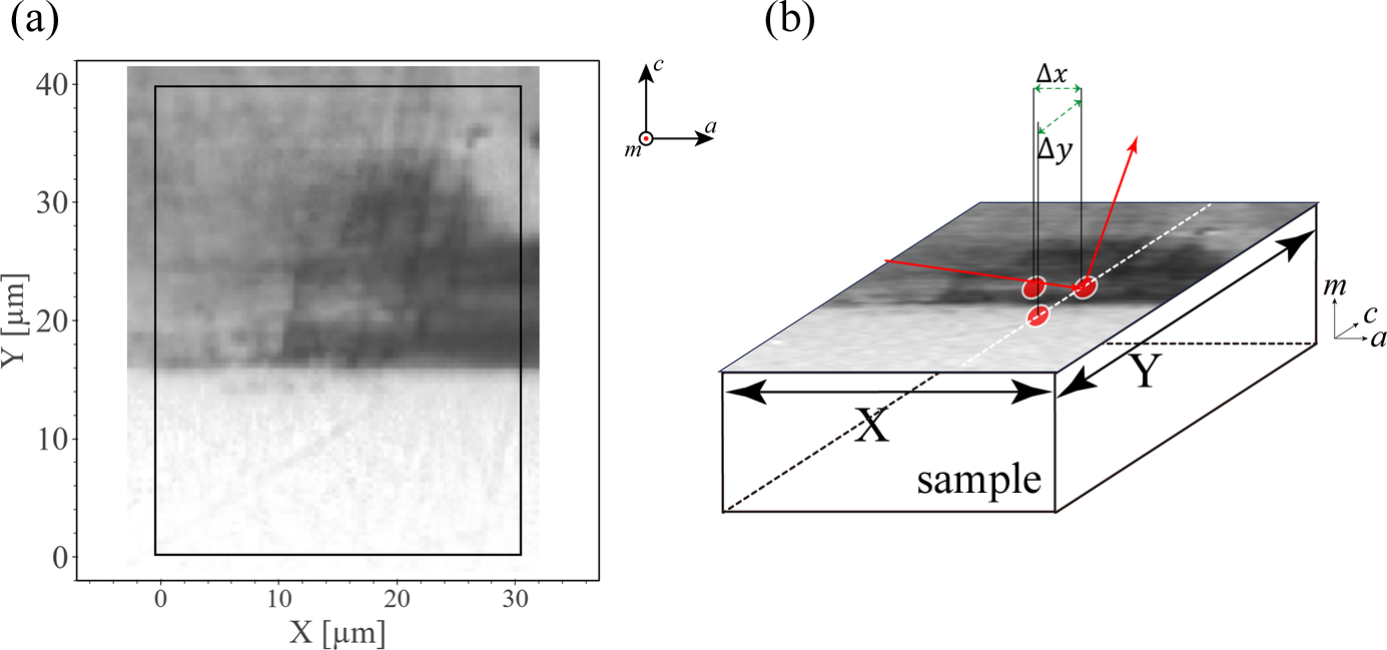
Sample and experiment setup. (*a*) Cross-sectional MPPL image of the HVPE GaN sample. The interface between the thick HVPE-grown film and the GaN substrate is located at around *Y* = 15 µm. The nanoXRD measurement area is marked by a rectangle. (*b*) Schematic of the diffraction geometry and the spatial step between sampling points of Δ*X* = Δ*Y* = 1 µm with an angular step of δω = 0.002°.

An epitaxial GaN sample was grown on a low-defect-density GaN substrate by HVPE. A nanoXRD experiment was conducted on a rectanglular area from the cross-sectional HVPE GaN sample (Fig. 1 ▸). Fig. 1 ▸ shows the boundary between the epitaxial layer and the substrate located around *Y* = 17 µm. The synchrotron X-ray beam (410 nm horizontally × 700 nm vertically) on the BL13XU beamline at SPring-8 was used for the experiments. Prior to the nanoXRD measurements, the sample cross section was mapped by multiphoton photoluminescence (MPPL) (Hamachi *et al.*, 2021 ▸; Hamachi *et al.*, 2023 ▸; Kim *et al.*, 1997 ▸; Tanikawa *et al.*, 2018 ▸; Zipfel *et al.*, 2003 ▸) to locate different crystal growth sectors and defective areas. The measurement area of nanoXRD has a size of *X* × *Y* = 30 × 40 µm (the *X* and *Y* axes are parallel to the *a* and *c* axes of the GaN crystal, respectively). To obtain 3D ω–2θ–φ mapping, 2D (2θ–φ) diffraction patterns were collected by a photon-counting 2D detector with an angular (ω) step of 0.002° in a range of 0.2° and a spatial step of 1 µm. We collected data sets from both symmetric 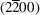 and asymmetric 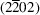 diffraction planes to ensure the input captured sufficient structural information. From each of the two diffraction planes, we measured 1271 sampling grid points on the bulk crystal.

### 2.2. Estimation of UMAP’s performance by synthesized data sets

**Figure 2:**
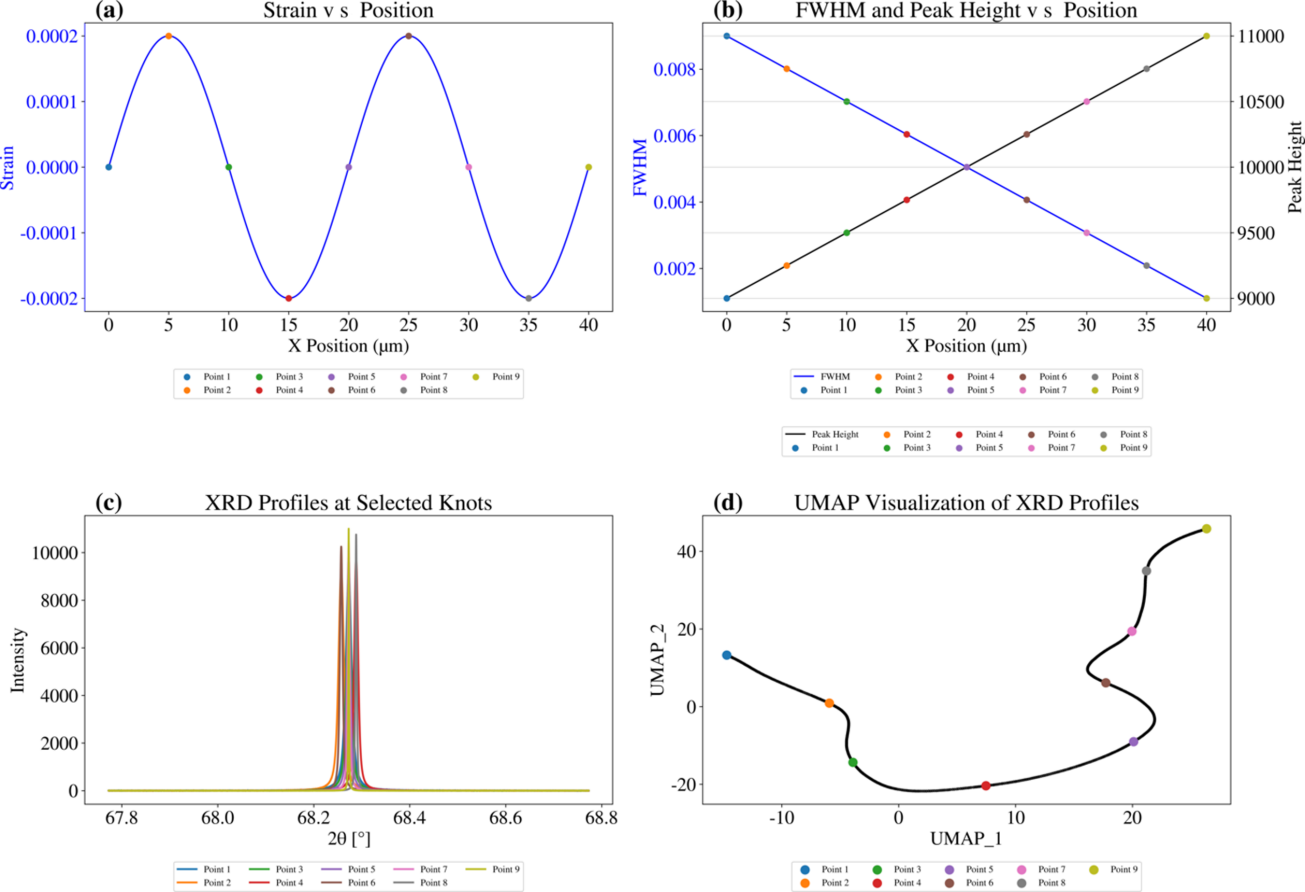
UMAP ability on synthesized 1D nanoXRD profiles. We synthesized the distribution of (*a*) strain and (*b*) FWHM and peak height versus *X* position. Selected points are marked in panels (*a*), (*b*) and (*d*). (*c*) Sampled XRD profiles at selected positions. (*d*) UMAP plot of reduced 1D nanoXRD profiles.

Although UMAP shows a faster and clearer clustering behavior in the field of biology compared with other dimensionality reduction algorithms such as PCA and *t*-SNE (Amir *et al.*, 2013 ▸; Van Der Maaten & Hinton, 2008 ▸), it remains unclear how the UMAP plot represents the crystal structural characteristics recorded in nanoXRD patterns, due to the lack of prior investigation. Before applying UMAP to practical nanoXRD data sets, we evaluated the performance of UMAP with synthesized 1D XRD data sets. To verify that UMAP can detect continuity changes in the XRD spectra, we designed a thought experiment by synthesizing a series of 1D XRD spectra, as shown in Figs. 2 ▸ and 3 ▸. We simulated the 2θ intensity spectrum from the 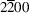 reflection with *a* = 3.189 Å. A continuously distributed strain field along the *X* direction with a sinusoidal shape was synthesized, as shown in Fig. 2 ▸(*a*). Correspondingly, the FWHM decreases monotonically and the peak height increases, as shown in Fig. 2 ▸(*b*). To capture position-dependent results, we extracted nine points at *T*/4 positions of the strain field. Their corresponding XRD profiles are shown in Fig. 2 ▸(*c*). Fig. 2 ▸(*d*) demonstrates that UMAP successfully captures the synthesized XRD spectrum’s continuously and monotonically evolving data structure.

**Figure 3:**
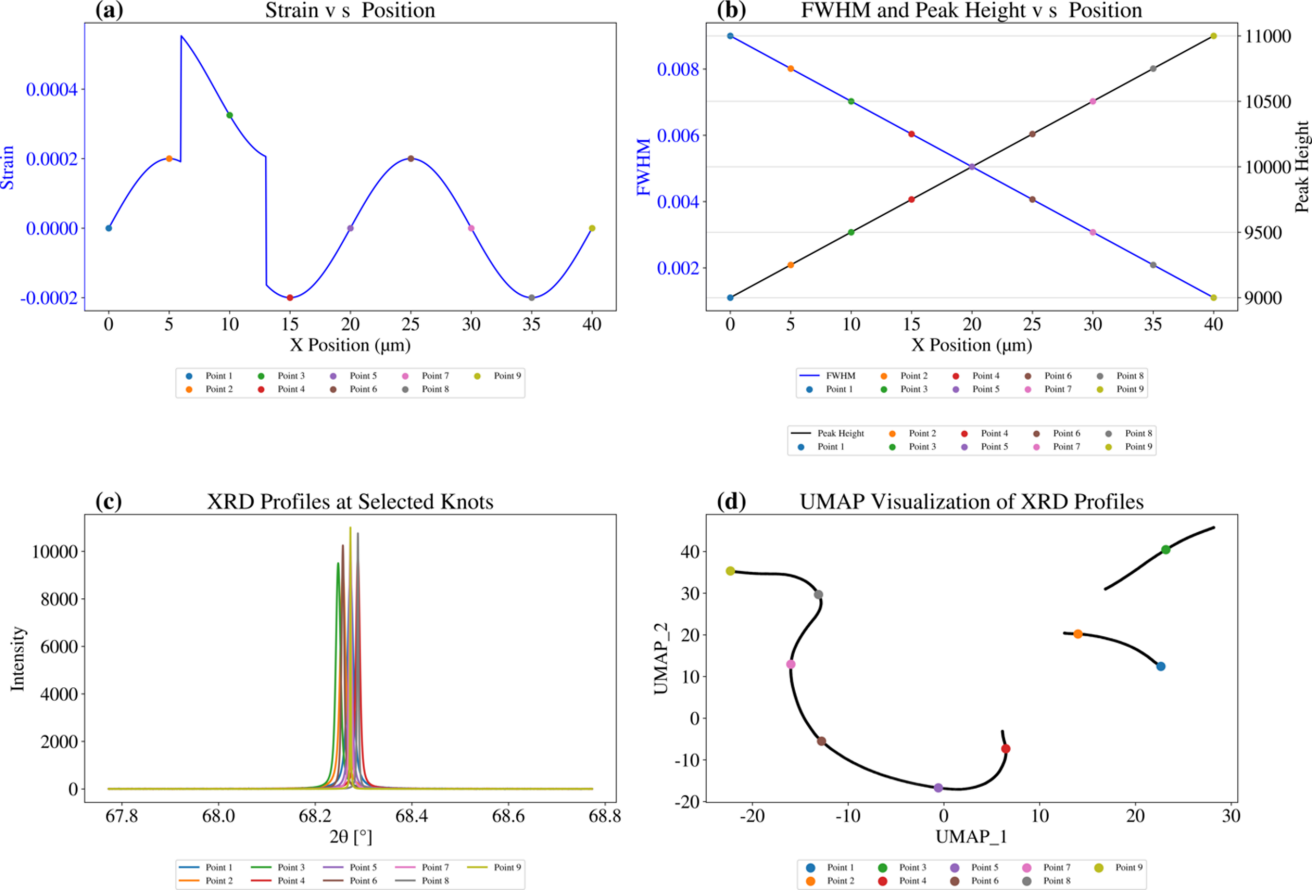
UMAP ability on synthesized 1D nanoXRD profiles with varying continuity. We synthesized the distribution of (*a*) strain and (*b*) FWHM and peak height versus *X* position. Selected points are marked in panels (*a*), (*b*) and (*d*). We insert a new strain state at *X* ∈ [6 µm, 13 µm]. (*c*) Sampled XRD profiles at selected positions. (*d*) UMAP plot of reduced 1D nanoXRD profiles.

To evaluate UMAP’s sensitivity to structural discontinuities, we introduced a strain field with an inserted segment, as illustrated in Fig. 3 ▸(*a*), while all other conditions remained unchanged. Fig. 3 ▸(*d*) shows that UMAP effectively detects the inserted segment as a distinct discontinuity, highlighting its ability to identify structural transitions on the basis of a continuity change in the data structure.

Note that these simulations serve solely as a proof-of-concept demonstration of UMAP’s performance; the simulated data themselves do not correspond to any practical conditions. In practical applications, the microstructural characteristics of each bulk crystal sample vary significantly and their effects on nanoXRD spectra are often highly complex and difficult to predict *a priori*.

### 2.3. UMAP analysis for nanoXRD data sets

**Figure 4:**
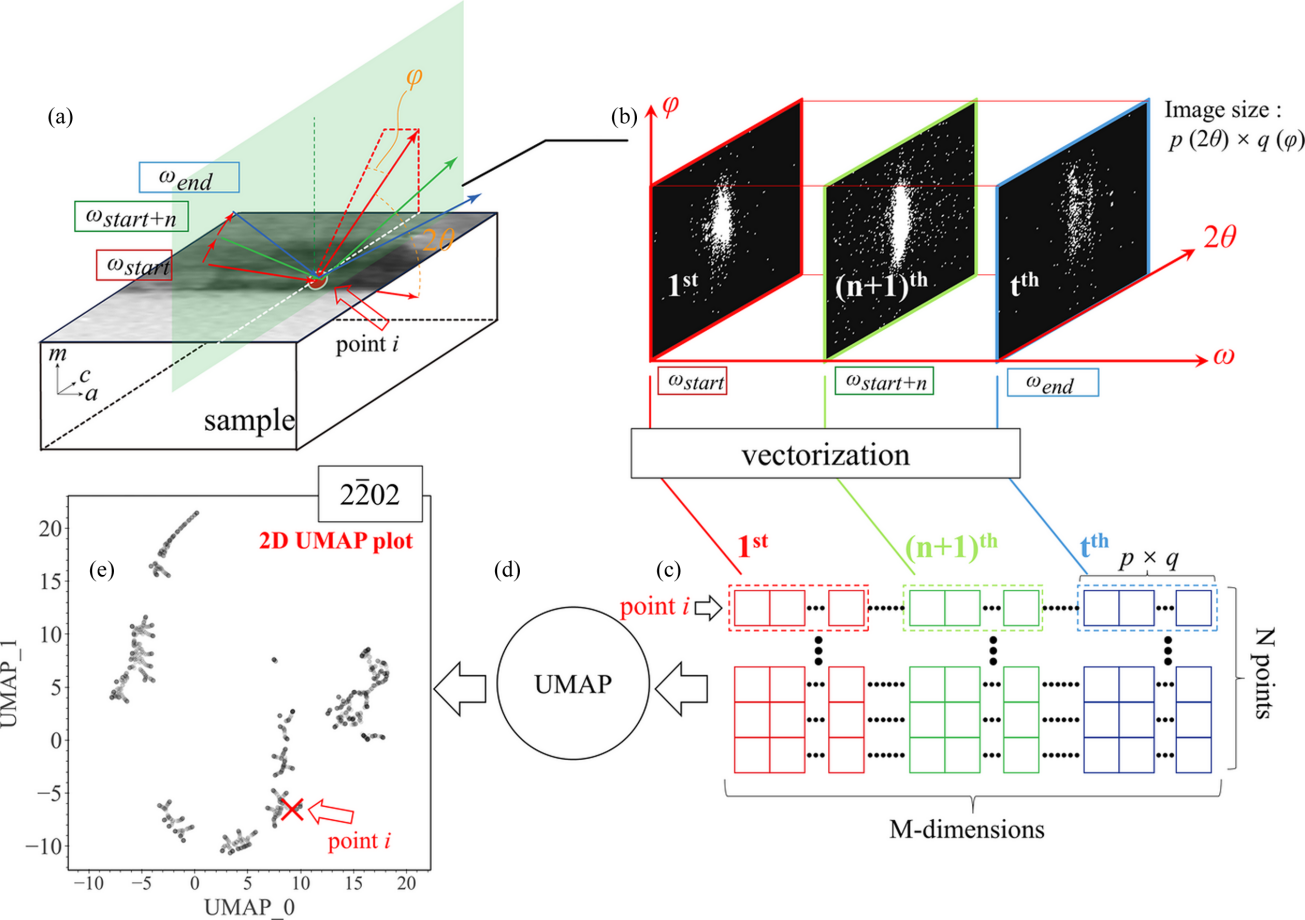
Schematic diagram of the workflow using UMAP to analyze a raw nanoXRD data set. (*a*) Schematic diagram of data acquisition in nanoXRD. (*b*) Each diffraction image corresponds to a specific angular position ω and sampling point *i*, as shown in panel (*a*), during the online experiment. For each diffraction image, the horizontal size *p* and vertical size *q* correspond to the angular range in the 2θ and φ directions, respectively. (*c*) Diffraction patterns from point *i* are transformed to a vector, the dimensions of which are determined by the size of the diffraction patterns. Similar processes are repeated for *N* sampling points. The data set prepared in (*c*) is fed into (*d*) the UMAP algorithm, which is (*e*) further embedded and clustered in a 2D space for visualization.

Fig. 4 ▸ is a schematic diagram of how we obtain and deal with the raw diffraction patterns using UMAP to reduce the input data into 2D space. First, as shown in Figs. 4 ▸(*a*) and 4 ▸(*b*), 3D ω–2θ–φ nanoXRD data are collected from the sample’s surface. At a sampling point, *e.g.* point *i*, the sample is rocked around the incident angle ω with an angular step of δω within the range of ω_start_ to ω_end_. So, at point *i*, the number of collected diffraction images is
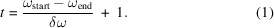


In this experiment, each sampling point contains *t* = 101 diffraction images, *i.e.* 2θ–φ axis diffraction images were collected at 101 scanning positions along the ω axis [Figs. 1 ▸ and 4 ▸(*b*)]. Figs. 4 ▸(*b*) and 4 ▸(*c*) show how *t* images, each of which has a horizontal size *p* and vertical size *q* corresponding to the angular ranges in the 2θ and φ directions, respectively, are flattened into a vector with dimensions of
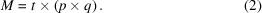


For the sampling area on the bulk crystal, by flattening the diffraction patterns from *N* = 1271 sampling grid points in this research, an array of 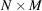 is finally obtained as input for UMAP [Fig. 4 ▸(*c*)].

By applying UMAP to the raw 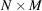 array data, the dimensionality of the data can be reduced from *M* to 2, *i.e.* non-numerical raw diffraction patterns are ‘embedded’ into a point in 2D numerical space. In the *M*-dimensional space, the distance between sampling points reflects the similarity (McInnes *et al.*, 2018*a* ▸; McInnes *et al.*, 2018*b* ▸). In other words, when the distance between two sampling points is small in the *M*-dimensional space, the difference in diffraction patterns from the two sampling points is small. With the help of a hypothesized manifold structure, UMAP reduces the data space from *M*-dimensional to 2D, while retaining the relative distance between sampling points to visualize the data structure (McInnes *et al.*, 2018*a* ▸; McInnes *et al.*, 2018*b* ▸). Fig. 4 ▸(*e*) shows the position of a random point *i* in the 2D UMAP plot, which embeds the 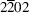 diffraction patterns from point *i* in Fig. 4 ▸(*a*). Note that the UMAP algorithm utilizes randomness to speed up the calculation, which means that different runs of UMAP can produce different results. Although UMAP is relatively stable, we have verified the robustness of UMAP’s results against the randomness and noise in Supplementary Note 1 in the supporting information.

### 2.4. UMAP representation of nanoXRD results

We will first compare the UMAP plots with conventional 1D diffraction profiles, which are straightforward and commonly used in research utilizing XRD. Then, we compare the UMAP plots with 2D nanoXRD results.

**Figure 5:**
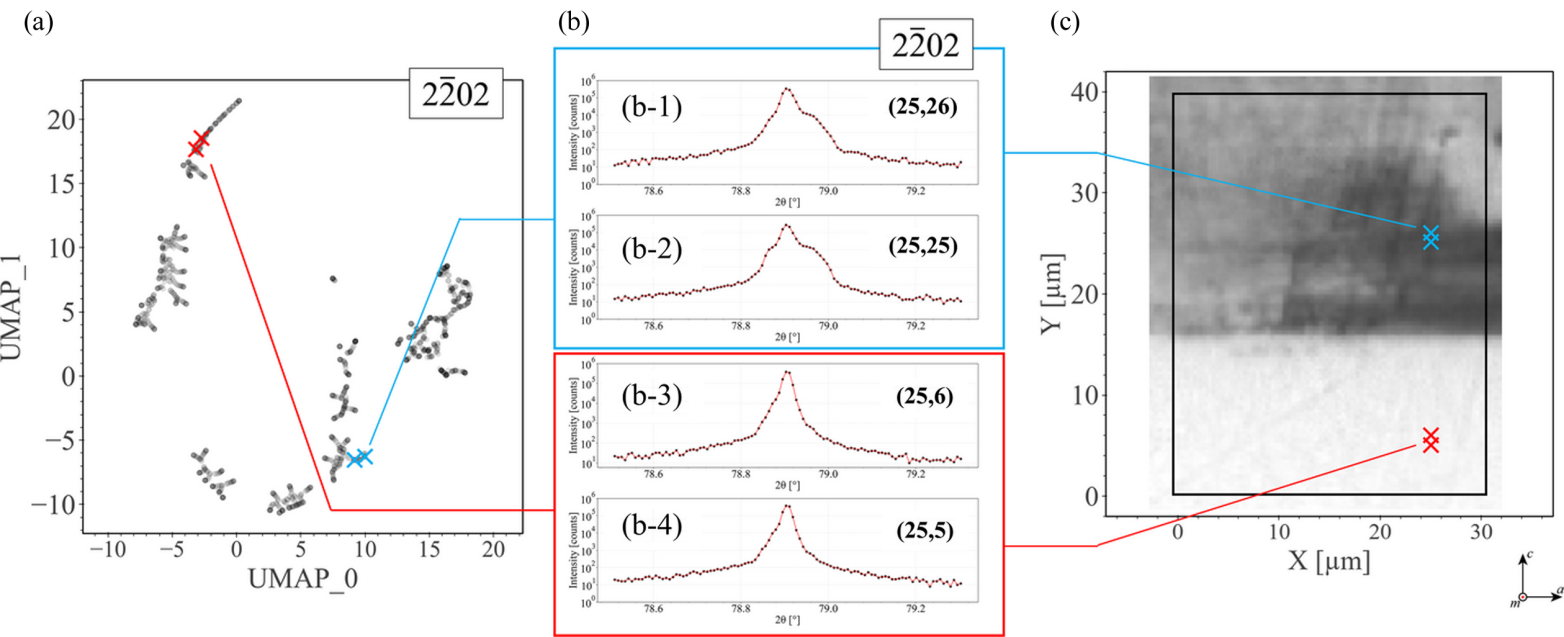
Comparison between UMAP results and conventional 1D diffraction profiles. (*a*) UMAP plot for 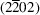 diffraction planes. Blue × markers correspond to the 2θ intensity profiles shown in panels (*b*-1) and (*b*-2), while red × markers correspond to those shown in panels (*b*-3) and (*b*-4). (*c*) Red and blue × markers show the corresponding diffraction positions in the sample.

Fig. 5 ▸ compares 2D UMAP results and several representative 1D profiles obtained from raw nanoXRD data. Although conventional 1D profiles can only partially represent the raw diffraction patterns, we can utilize 1D profiles to conduct initial research on the properties of clustering made by UMAP plots. In Figs. 5 ▸(*a*) and 5 ▸(*c*), × markers indicate several sampling points in the UMAP plot and the corresponding diffraction positions in the sample. When the sampling points are located close to each other in the sample, *e.g.* the blue × markers in Fig. 5 ▸(*c*), the 2θ intensity profiles are similar, as shown in panels (b-1) and (b-2), reflecting the similarity in the structures. Meanwhile, Fig. 5 ▸(*a*) shows that the distance between the blue × markers in the 2D UMAP plot is also small. Similar results are observed for a different set of points shown by the red × markers in Fig. 5 ▸(*c*) [Figs. 5 ▸(*b*-3) and 5 ▸(b-4)]. Due to the significant differences between the diffraction profiles of the blue and red × markers shown in Fig. 5 ▸(*b*), the relative distance between the blue and red × markers in the UMAP plot [Fig. 5 ▸(*a*)] is significant. The relationship between the profiles’ similarity and distances in the UMAP plot shown in Fig. 5 ▸ indicates that the distance between sampling points in the reproducible 2D UMAP plot gives a direct visualization of their similarity. Note that the UMAP plots shown in Fig. 5 ▸(*a*) embed 3D ω–2θ–φ intensity results instead of 1D ω or 2θ intensity profiles. Although the 1D XRD profiles lose much information compared with the raw 3D patterns, the results in Fig. 5 ▸(*b*) suggest that, to some extent, 1D profiles partially represent the similarities between raw 3D data sets. We also emphasize that the asymmetric shapes of the 1D 2θ intensity profiles shown in Fig. 5 ▸(*b*) do not match an ideal fit by Gaussian functions, which means that we cannot easily extract structural information from conventional methods, thereby validating the efficiency and necessity of utilizing UMAP.

A comparison between the UMAP plots and conventional 1D ω intensity profiles implies the importance of assistance from UMAP, especially when one wants to analyze crystal structure changes or classify crystal growth sectors according to the similarities between diffraction patterns, and particularly when the sample has a complex structure and an obscure 1D diffraction spectrum.

**Figure 6:**
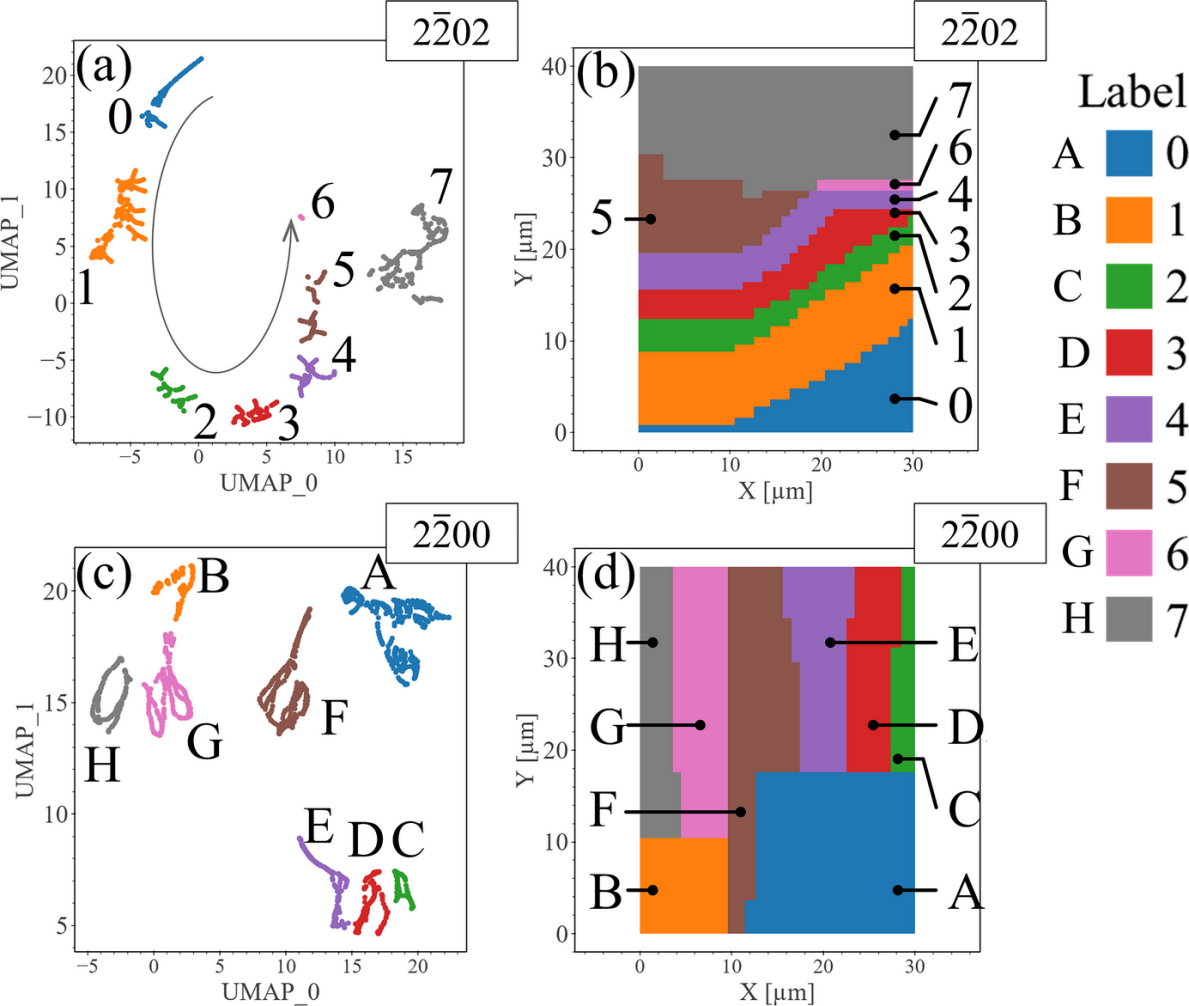
Clustering results of the 2D UMAP plots. Results for (*a*) 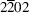 and (*c*) 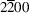 are listed. Clusters in panels (*a*) and (*c*) are colored according to their labels. The elliptical arrow curve represents a guide to the eye to illustrate the continuity of the cluster distribution. (*b*), (*d*) Mapping of the UMAP clusters into the nanoXRD measurement area. Mapping colors in panels (*b*) and (*d*) are linked to the clusters in panels (*a*) and (*c*), respectively.

Since similar diffraction patterns are embedded to be located close to each other in the UMAP plots, we can further categorize the sample’s structure using clusters in the UMAP plots, as shown in Fig. 6 ▸ for the 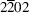 and 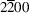 diffraction patterns. Clusters were classified with the help of the agglomerative hierarchical clustering method (Murtagh & Contreras, 2012 ▸) and labeled for convenience in the discussions below: labels for 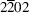 are from 0 to 7 [Fig. 6 ▸(*a*)] and those for 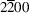 are from A to H [Fig. 6 ▸(*c*)]. More discussion on the selection of the number of clusters can be found in Supplementary Note 2. The measurement area is colored according to the clusters in the UMAP plots [Figs. 6 ▸(*b*) and 6 ▸(*d*)].

When we compare the results for 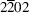 and 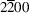 and look at the relationship between clusters by observing the relative positions of the clusters in Fig. 6 ▸, the distribution of the clusters for 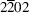 differs from that for 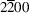. The clusters 0 to 6, *i.e.* excluding cluster 7, are elliptically distributed in the UMAP plot [see the guide to the eye in Fig. 6 ▸(*a*)], suggesting the detection of continuity of structures often seen in UMAP analysis (Becht *et al.*, 2019 ▸; Zheng *et al.*, 2023 ▸), while the relative positions between the clusters in Fig. 6 ▸(*c*) seem more incoherent than those in Fig. 6 ▸(*a*). A comparison between the UMAP plots and the map of clusters on the measurement area implies more differences: clusters 0 to 7 in Fig. 6 ▸(*b*) are distributed approximately along the *c* direction of the sample, while clusters C to H in Fig. 6 ▸(*d*) are distributed along the *m* direction. This difference is probably caused by the fact that the *c* plane information dominates the 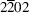 diffraction pattern, while the 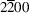 diffraction pattern reflects *m* plane information, although both include the *m* plane information.

Note that the cluster derived from UMAP does not fully coincide with the contrast changes in the MPPL images. For example, the boundary of the region labeled 7 in the 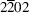 UMAP plot located at around *Y* = 27 µm [Fig. 6 ▸(*b*)] is not apparently suggested by the MPPL image [Fig. 5 ▸(*c*)], whereas the region labeled A in Fig. 6 ▸(*d*) has a boundary around *Y* = 17 µm which coincides with the boundary from the MPPL image [Fig. 5 ▸(*c*)]. We believe this difference is due to the following reason. Unlike XRD, which directly records structural information from the diffraction volume, the intensity contrast in the luminescence image depends on the band structure, which can be influenced by factors such as strain, impurities and defects. While dislocations or impurities are known to distort the luminescence spectrum in GaN (Hamachi *et al.*, 2021 ▸; Kim *et al.*, 1997 ▸; Tanikawa *et al.*, 2018 ▸; Zipfel *et al.*, 2003 ▸), it is difficult to establish a clear relationship between the MPPL spectrum and potential defects or stress distribution in the measurement area. As a result, the luminescence intensity change may not directly align with the distribution of defects or stress in the measurement area. Consequently, we think the MPPL data do not capture the structural changes in the sample as thoroughly and clearly as the XRD data do.

**Figure 7:**
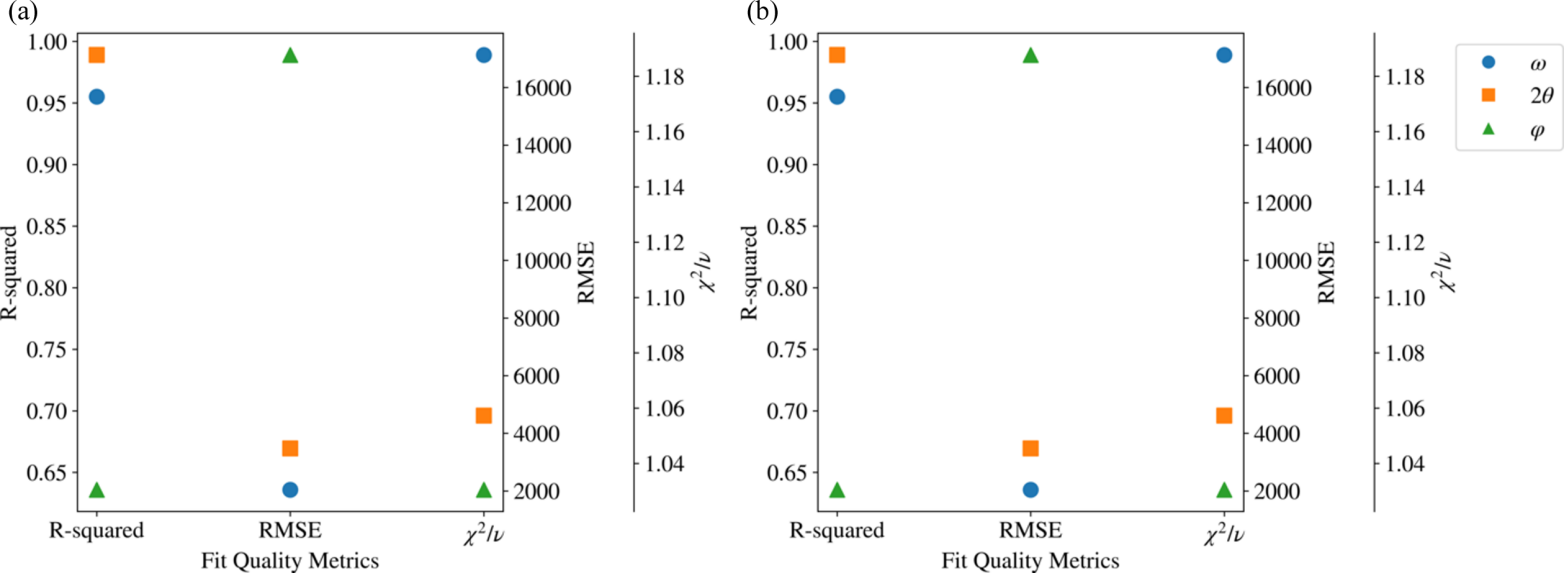
Fit quality metrics for traditional Gaussian fits. Results are calculated for (*a*) 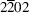 and (*b*) 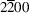. For each plot, averaged metrics calculated for ω, 2θ and φ intensities are colored accordingly.

As a reference, we also constructed crystal structure maps of lattice constants *a* and *c* and lattice tilting fluctuations derived from the FWHM of ω using the conventional method (see Fig. S5 in Supplementary Note 3) However, although these structure maps show some distribution of structural components, the crystal information is not straightforwardly understandable. The Gaussian fit quality on the nanoXRD data set obtained in this work is evaluated by various metrics and illustrated in Fig. 7 ▸, which shows the averaged value of *R*^2^, root-mean-square error (RMSE) and reduced χ^2^ values. The relatively high reduced χ^2^ values for the ω intensity suggest a low fit quality, while the fit quality of the 2θ intensity profiles is relatively high (*R*^2^ close to 1, with low RMSE and low reduced χ^2^ values). Meanwhile, low *R*^2^ and high RMSE values reveal a poor match between the raw and fitted data in the φ direction. Fig. 7 ▸ quantitively suggests that the quality of traditional Gaussian fitting is not stable and reliable in this research. Further discussion of the fit quality can be found in Supplementary Note 3.

More than the uncertain fitting of asymmetric profiles, we believe the unintelligibility of results by the conventional method is due to incomplete information obtained from the 1D diffraction spectrum. Neither the peak position nor width of the 1D diffraction spectrum can wholly and solely describe the evolution of crystal structure. In contrast, raw 3D data sets provide richer structure information, such as the broadening direction and shape of diffraction spectra, which can enhance the understanding of crystal evolution. Remarkably, UMAP can fully detect diffraction details, including peak position, width and broadening, from the raw 3D data sets simultaneously, influencing the clustering results in UMAP plots.

### 2.5. Two-dimensional nanoXRD data analysis assisted by clusters from UMAP

**Figure 8:**
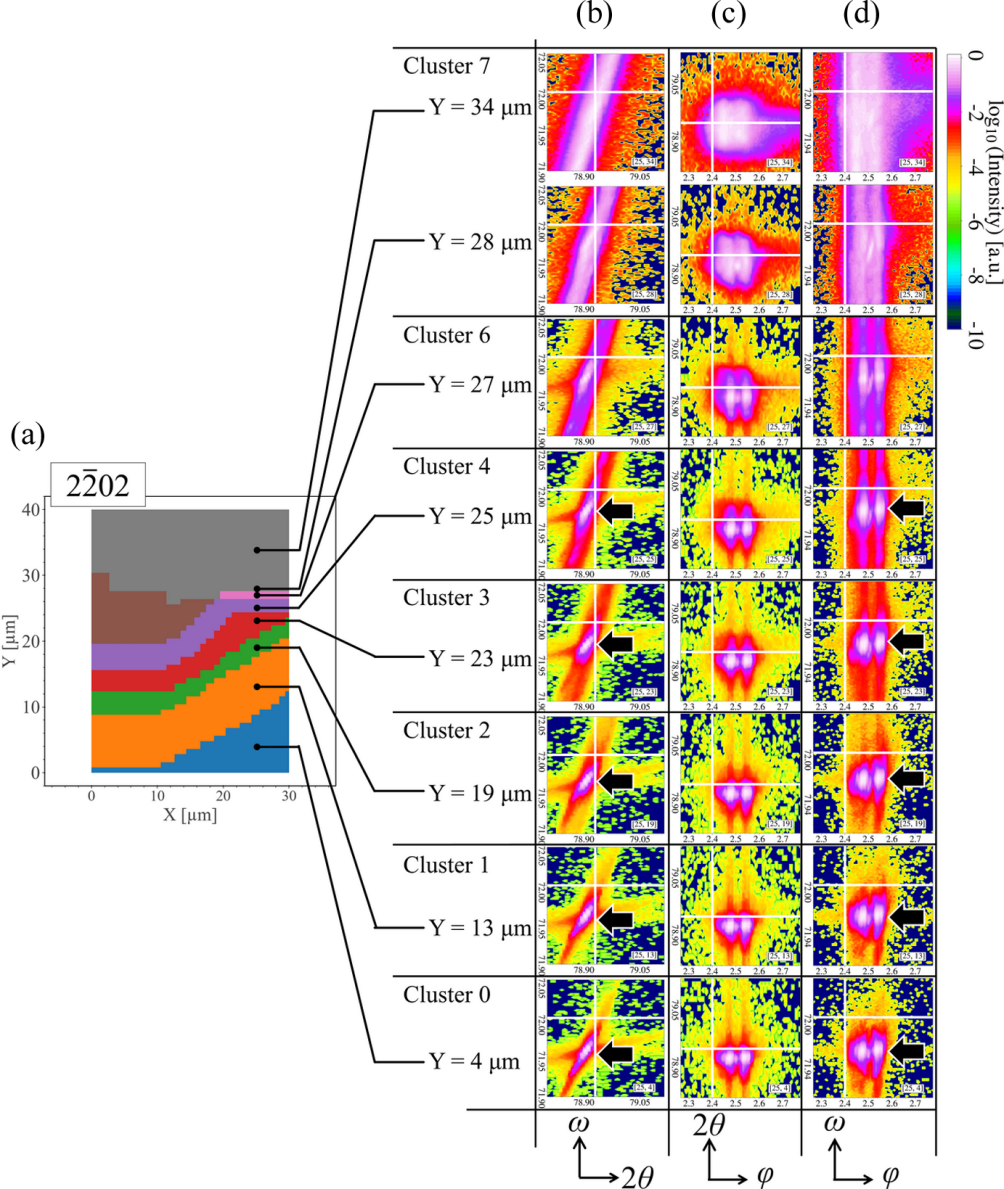
(Right) Matrix of representative 2D 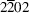 intensity profiles at *X* = 25 µm positions along the *Y* direction in (*a*) the UMAP cluster map. (*b*)–(*d*) Columns of raw 3D diffraction patterns projected onto 2D space. The coloring of each profile is based on logarithmic values of intensity. Column (*b*) in the matrix is for ω–2θ space, column (*c*) is for 2θ–φ space and column (*d*) is for ω–φ space. Each row of the profiles is labeled according to the UMAP cluster map shown in panel (*a*). For each image, a white crosshair is used as a guideline to help determine the positions of the diffraction peaks, and black arrows are used to indicate the peak positions. Because of the zone plate in the optical system of nanoXRD, two separate peaks are observed along the φ direction. The values of φ are shown to be relative since a reference diffraction point along the φ direction is not given.

To utilize the UMAP cluster map for extracting physical information on crystal structures, we compare the map with some representative 2D intensity profiles taken from respective clusters. Even though we have stated that 1D ω or 2θ intensity profiles can represent the similarities between 3D raw data sets to some extent, the loss of information due to the processing from raw data to 1D profiles may lead to mistakes in crystal structure analysis. To avoid this and fully understand the clustering behavior by UMAP, we listed the 2D intensity profiles of different projections from the 3D ω–2θ–φ diffraction results. Fig. 8 ▸ shows a matrix of 2D 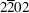 intensity profiles taken from different *Y* positions at *X* = 25 µm, with corresponding labels as shown in Fig. 6 ▸(*b*).

In the left-hand column of the matrix [Fig. 8 ▸(*b*)], dark arrows indicate that the diffraction peak in the ω–2θ intensity profile shifts continuously in the ω direction as the sampling point changes sequentially from clusters 0 to 4. Such continuous shifting coincides with the aforementioned elliptical distribution of these clusters on the UMAP plot of Fig. 6 ▸(*a*), which suggests that UMAP can probably visualize continuity in the data and embed the diffraction patterns into the same cluster according to their similarity.

Compared with cluster 4, the peak area in cluster 6 is considerably broadened. The peak abruptly broadens in the ω direction in cluster 7, regardless of the small distance of about 1 µm between the sampling points in clusters 6 and 7. The abrupt broadening in cluster 7 explains its isolation from the elliptical and continuous distribution collectively formed by clusters 0 to 6. Such a continuous shift in the peak positions, *i.e.* an increase in ω values as the sampling points change from clusters 0 to 4, and a sudden broadening in the ω direction in cluster 7, are also observed in the ω–φ intensity profiles [Fig. 8 ▸(*d*)].

In contrast, in the 2θ–φ profiles of Fig. 8 ▸(*c*), the change in the peak shape in cluster 7 is not as evident as those in the ω–2θ and ω–φ profiles. Unlike the continuous shift and the considerably elongated shape in the ω direction of the peak, the shift and broadening of the peak in both 2θ and φ directions are limited. This fact suggests that the crystal structure changes reflected in the 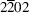 diffraction patterns are dominated by lattice plane tilting.

**Figure 9:**
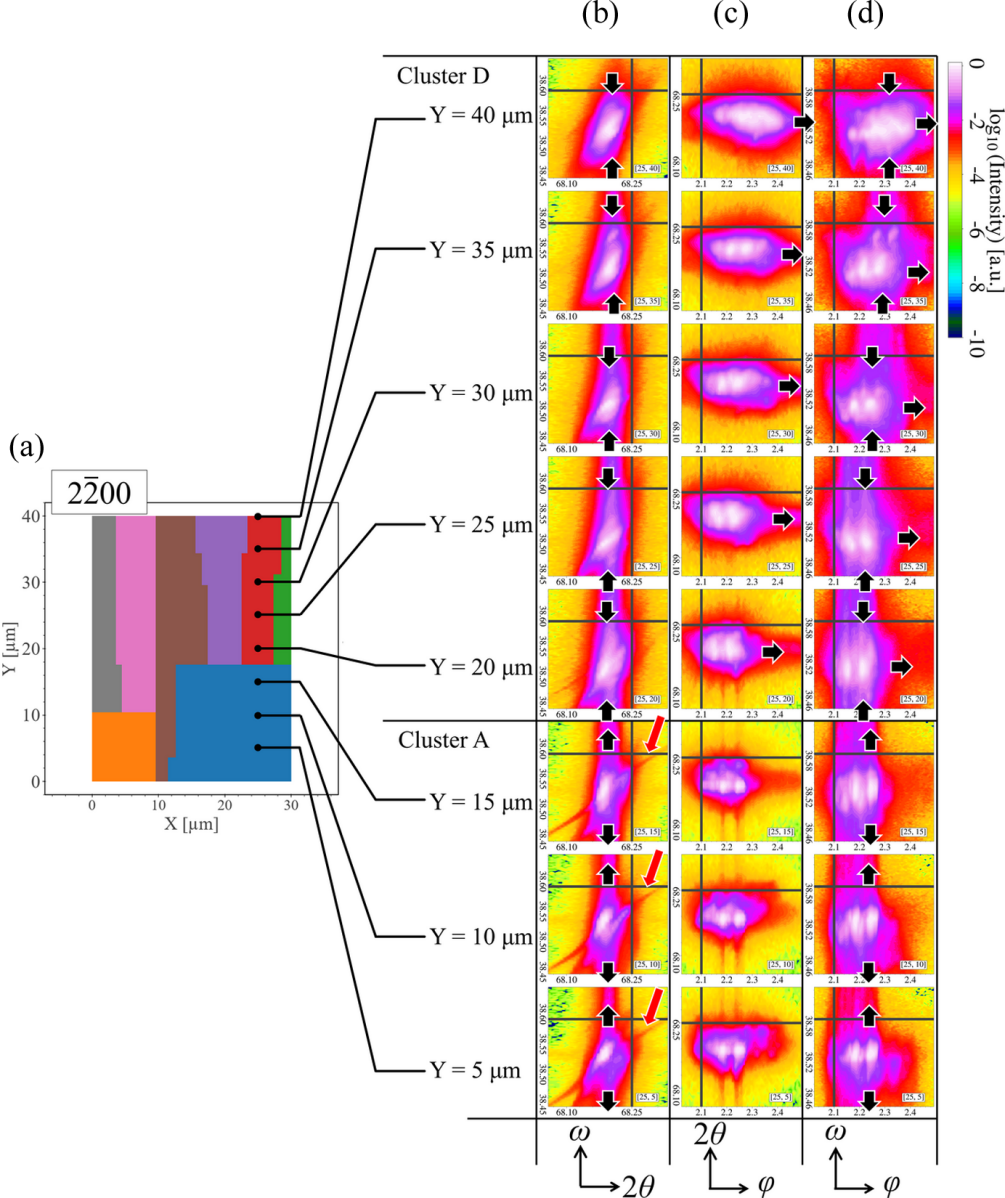
(Right) Matrix of representative 2D 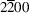 intensity profiles at *X* = 25 µm positions along the *Y* direction in (*a*) the UMAP cluster map. (*b*)–(*d*) Columns of raw 3D diffraction patterns projected onto 2D space. The coloring of each profile is based on logarithmic values of intensity. Column (*b*) in the matrix is for ω–2θ space, column (*c*) is for 2θ–φ space and column (*d*) is for ω–φ space. Each row of the profiles is labeled according to the UMAP cluster map shown in panel (*a*). For each image, a dark crosshair is used as a guideline to help determine the positions of the diffraction peaks, and dark arrows in panels (*b*) and (*d*) indicate how the diffraction patterns shrink or broaden in the ω direction, while dark arrows in panel (*c*) emphasize the movement in the φ direction. Red arrows in panel (*b*) mark the position of a minor structure near the peak. Because of the zone plate in the optical system of nanoXRD, two separate peaks are observed along the φ direction. The values of φ are relative since a reference diffraction point along the φ direction is not given.

We further compared the UMAP cluster map and representative 2D 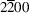 intensity profiles at *X* = 25 µm. As shown in Fig. 9 ▸, there are only two clusters along the *Y* axis, which differs from the six vertically distributed clusters seen in Fig. 8 ▸ for the 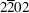 reflection. It is apparent that the features of the diffraction patterns of cluster A are distinguishable from those of the diffraction patterns of cluster D. For example, in diffraction patterns from cluster A, only minor changes have occurred with increasing *Y* position: an appearance of minor structure near the peak marked by red arrows in the ω–2θ profiles and a slight broadening of the diffraction peaks in the ω direction with negligible shifting in the φ direction. In contrast, in cluster D, the diffraction peaks exhibit notable variation with a shift in the φ direction towards higher angles and a narrowing in the ω direction with negligible shifting in the 2θ direction (dark arrows in Fig. 9 ▸).

### 2.6. Interpretation of structure categorization by UMAP

**Figure 10:**
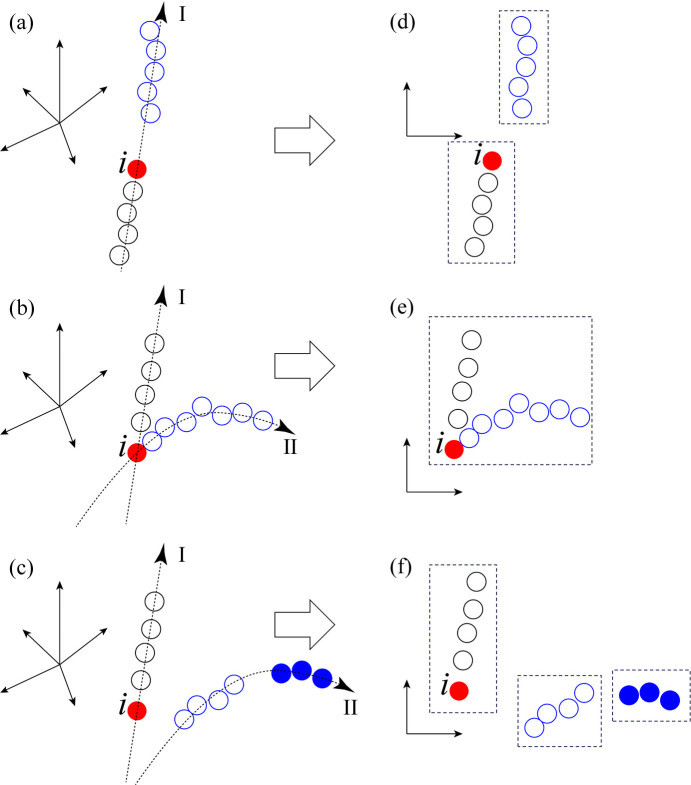
Schematic models for distribution of sampling points. (*a*)–(*c*) Three types of model around a point *i* in high-dimensional space, and (*d*)–(*f*) the corresponding hypothesized clustering results obtained by the UMAP analysis, in which dashed rectangles mark the positions of the clusters. The obtained 2D UMAP plots shown in Fig. 6 can be modeled by these distributions. For example, the continuous distribution of clusters 0 to 5 observed in Fig. 6(*a*) is modeled here by panels (*a*) and (*d*), the branches observed in most clusters are modeled here by panels (*b*) and (*e*), and the incoherent distribution of the clusters observed in Fig. 6(*c*) can be modeled here by panels (*c*) and (*f*).

This section discusses how UMAP categorizes diffraction patterns in terms of the similarity and continuity of their data structures. In UMAP, the similarity is represented by the relative distances between sampling points in a high-dimensional Euclidean space, while the continuity can be understood from the perspective of the distribution of sampling points. Three representative types of sampling point distribution in high-dimensional Euclidean space are exemplified in Figs. 10 ▸(*a*) to 10 ▸(*c*), and related results of the 2D UMAP plot are shown in Figs. 10 ▸(*d*) to 10 ▸(*f*). Here, the nearest neighbors of point *i* have data structures similar to that of point *i*, and different distribution branches marked by dashed lines I and II represent different continuities of the change in data structure. The sampling points in Fig. 10 ▸(*a*) are distributed along branch I with a large gap, which results in the sampling points being classified into two clusters divided by the gap, as shown in Fig. 10 ▸(*d*). If the data structure has a different continuity, branch II is introduced, as shown in Fig. 10 ▸(*b*). Although we expect clusters to be classified on the basis of continuity, the high similarity between neighbors around point *i* prevents them from separating and causes a single cluster, as shown in Fig. 10 ▸(*e*). On the other hand, if the similarity is low between neighbors in different branches or in the same branch, as shown in Fig. 10 ▸(*c*), we can expect a rather incoherently clustered result, as shown in Fig. 10 ▸(*f*).

The scenario depicted in Fig. 10 ▸ implies that not only does the distribution of clusters reflect the continuity of the data structure, as shown in Fig. 6 ▸(*a*), but also the relative positions between sampling points within the cluster reflect their continuity. As an example, the results of the UMAP analysis focusing solely on cluster D are shown in Figs. S8 and Fig. S9 in Supplementary Note 4. Even within a cluster, diffraction patterns are found to be distributed according to their continuity, which again validates that both similarity and continuity are crucial factors in analyzing data structures.

### 2.7. Assessment of the crystal structure assisted by UMAP clusters

With the help of the data structure, including similarity and continuity, supplied by the UMAP analysis, the structural characteristics of the sample are discussed. Due to the inherent continuity of a perfect crystal structure during epitaxial growth, the obtained diffraction data are expected to exhibit unique continuity. However, unavoidable structural imperfections can lead to changes in this continuity. Such imperfections may include deformations in the unit cell or interplanar spacing, which are related to defects and external strain introduced by changing crystal growth modes or impurity incorporation. These changes, in turn, affect the diffraction patterns. It is also presumed that diffraction patterns of structures experiencing stable and uniform deformation exhibit consistent and continuous changes. For instance, a continuous peak shift may relate to a consistently increasing strain introduced by a persistent strain source, such as dislocations. Therefore, the different distributions of diffraction patterns in the high-dimensional space probably imply various structural deformations.

On the basis of the above evaluation criteria, we discuss the structural changes in the present sample with the help of Figs. 8 ▸ and 9 ▸ in more detail. Firstly, the continuous distribution of clusters in Fig. 6 ▸(*a*) infers a gradual shift of the 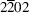 diffraction peak in the ω direction up to around *Y* = 27 µm, as shown in Fig. 8 ▸, indicating that the *c* planes are continuously tilted. This continuous change with a relatively constant rate is likely to be due to two contributing factors. The first factor is the long-range effect of stress originating from regions beyond the measurement range, probably due to the inhomogeneity of defect distribution in the sample, which causes the tilting of *c* planes observed in clusters 0 to 6. The second factor is related to the bright/dark contrast change around *Y* = 17 µm observed in the MPPL image [Fig. 5 ▸(*c*)], of which more details will be discussed later from the point of view of the 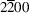 diffraction data.

**Figure 11:**
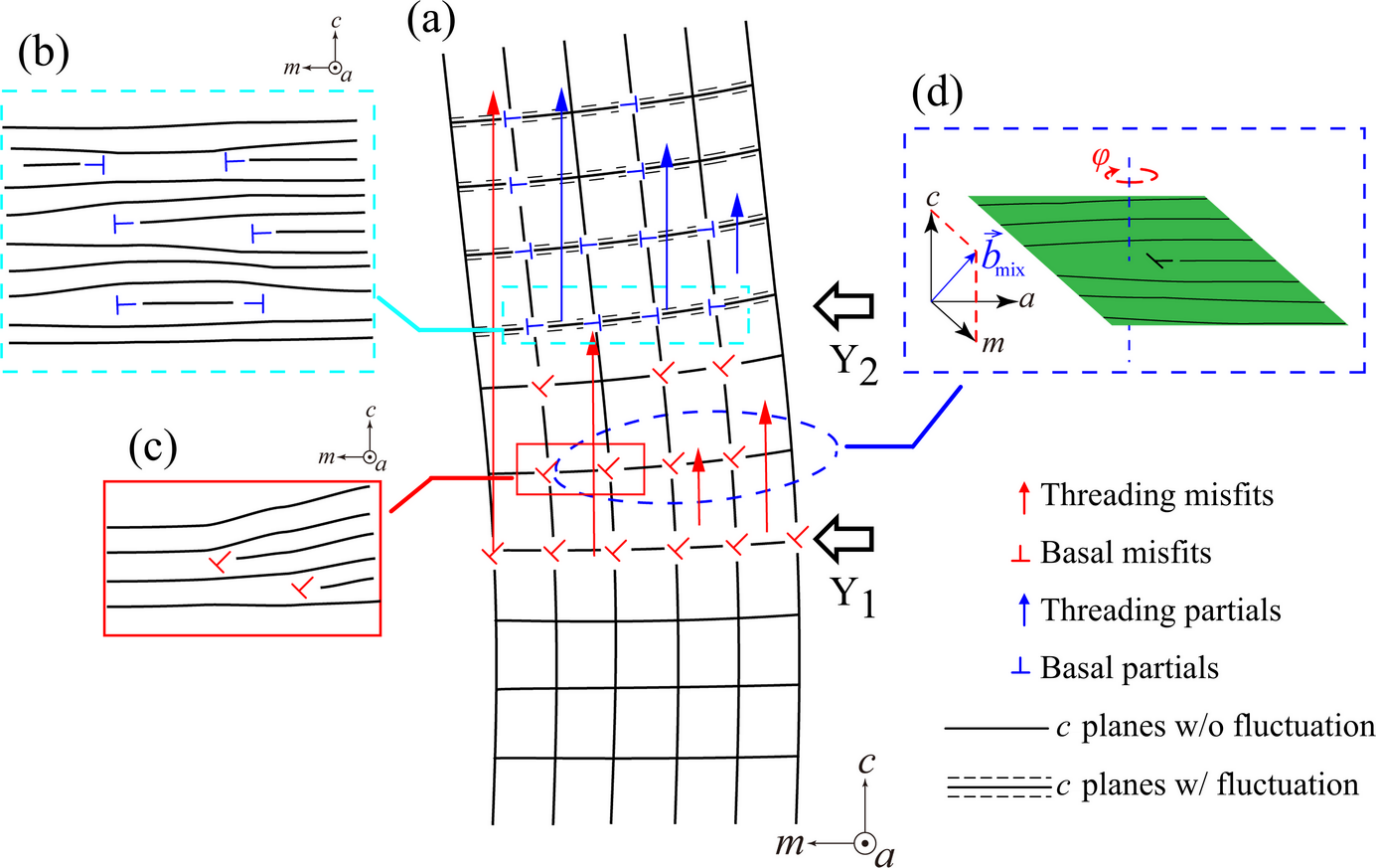
Schematic diagrams of the crystal structure. (*a*) Sketch of the crystal structure relating to the *c* planes around the positions of *Y* = 17 µm (*Y*_1_) and *Y* = 27 µm (*Y*_2_) with the symbols representing the type and distribution of defects. The structure morphologies correspond to different defects, including (*b*) stacking faults, (*c*) misfit dislocations of basal planes and (*d*) threading segments of the misfit dislocation.

On the other hand, for cluster 7 in Fig. 8 ▸ (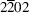 reflection), there is a sudden broadening of the diffraction peak in both ω and 2θ directions around the boundary at *Y* = 27 µm, suggesting a *c*-plane fluctuation. This sudden change in cluster 7 is probably caused by a new deformation source, such as stacking faults. Fig. 11 ▸(*b*) illustrates that stacking faults introduced in the *c* planes induce significant fluctuation of the *c* planes. The fact that stacking faults introduced in the *c* plane have only a limited effect on *m*-plane fluctuations explains the results for the 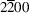 diffraction data in Fig. 9 ▸, that is, the absence of a boundary at *Y* = 27 µm in the UMAP cluster map for 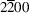.

Meanwhile, for the 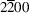 reflection characterizing the *m*-plane structure (Fig. 9 ▸), there is a significant and discontinuous difference in the diffraction peak’s shape and positions in the ω direction across the boundary around *Y* = 17 µm, which reflects the incoherent distribution of clusters A and D in Fig. 6 ▸(*c*). Since the formation of the gap between these clusters can be attributed to the different continuities, the *m* planes above *Y* = 17 µm are deduced to suffer from another type of defect that has limited influence on the *m* planes below *Y* = 17 µm. As a main source of this defect, we can consider misfit dis­locations. As shown in Fig. 9 ▸, a peak shift towards higher φ positions was observed at *Y* > 17 µm. We attribute this shift to the introduction of misfit dislocations in that region. We also observed a minor variation in φ towards the lower position, but this may be attributed to the effect of X-ray penetration and the appearance of stacking faults (see Supplementary Note 5 and Figs. S10 to S13 for a detailed discussion). The different trends in peak shifts in the φ direction imply various structural deformations. Since dislocations behave as non-radiative recombination centers, the appearance of dark contrast at the boundary observed in Fig. 5 ▸(*c*) probably signals the existence of misfit dislocations. The threading components of the misfit dislocations and partial dislocations associated with stacking faults generally have a mixed character and cause both tilting and twisting effects on the *m* plane, as illustrated in Fig. 11 ▸(*d*). Dislocations only induce a local change in lattice plane spacing around the dislocation core, which explains the minor change observed in the diffraction patterns in the 2θ direction. Note also that, since the degree of change in structural morphology depends on the density of defects, the gradual movement of diffraction patterns in the φ direction indicates a varying density of dislocations, which probably originates from the annihilation or generation of dislocations in the highly distorted area.

### 2.8. Interpretation of local crystal structure evolution assisted by data continuity

Here, we conduct a more detailed analysis of the local structural variations using the 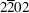 diffraction data. In the previous section, it was concluded that clusters 0 to 6 in Fig. 6 ▸(*a*) have a similar continuity attributed to the long-range effect of stress originating from regions beyond the measurement range and the boundary around *Y* = 17 µm. With the help of the aforementioned discussions, we now demonstrate that within cluster 7 as a representative example, the relative positions between sampling points reflect the continuity, and different kinds of structural imperfections induce the different continuities observed in the UMAP plot.

**Figure 12:**
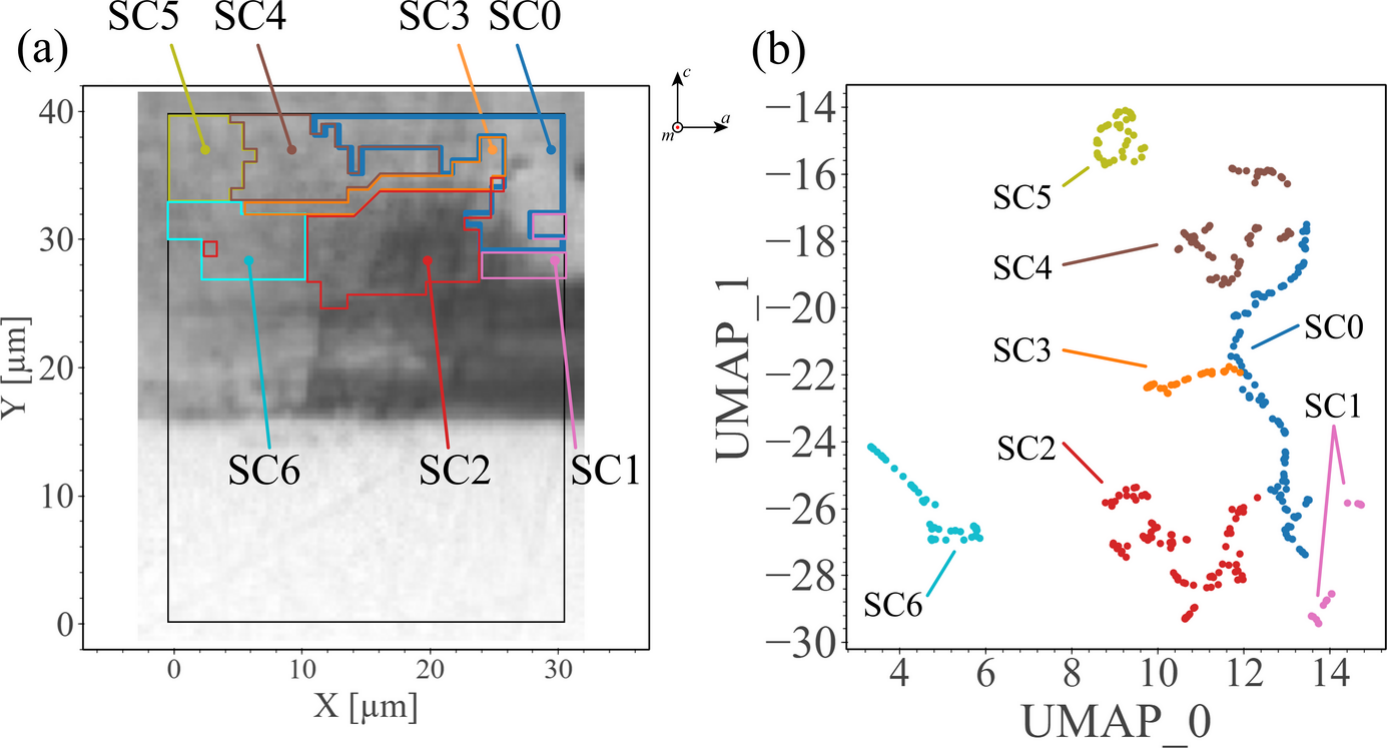
Analysis of continuity of the 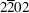 diffraction data. (*a*) MPPL image of the sample with the overlaid map of secondary clusters in panel (*b*). (*b*) UMAP plot of the cluster 7 area only, where the tree-like structure’s stem and branches named SC0 to SC6 are manually colored.

Fig. 12 ▸(*b*) shows the UMAP plot only containing the data of cluster 7. A tree-like data structure is observed with a manually colored stem and branches, named secondary clusters (SCs). As shown in Supplementary Note 6, to achieve an intuitive segmentation that reflects the tree-like data structure, we employed manual (visual) classification of SCs, acknowledging that this approach inherently introduces a degree of subjectivity. Specifically, (i) we examined the data structure across different UMAP parameter settings, (ii) we initially applied several clustering techniques to obtain preliminary clusters for identifying distinct branches, as the relatively weak connections between the main stem and branches can guide cluster boundaries, and (iii) after establishing these branches, we ensured the main stem remained as long and thin as possible. The distribution of sampling points corresponding to different parts of the UMAP plot is marked in Fig. 12 ▸(*a*). Interestingly, the bright/dark contrast in MPPL images is roughly related to the distribution of different SCs, *e.g.* the bright area in the upper right-hand corner of Fig. 12 ▸(*a*) corresponds to SC0, while SC2 covers most of the dark contrast in cluster 7. The correspondence between the MPPL image and the SC distribution provides additional evidence that SC0 and SC2 represent crystal structures with different characteristics.

**Figure 13:**
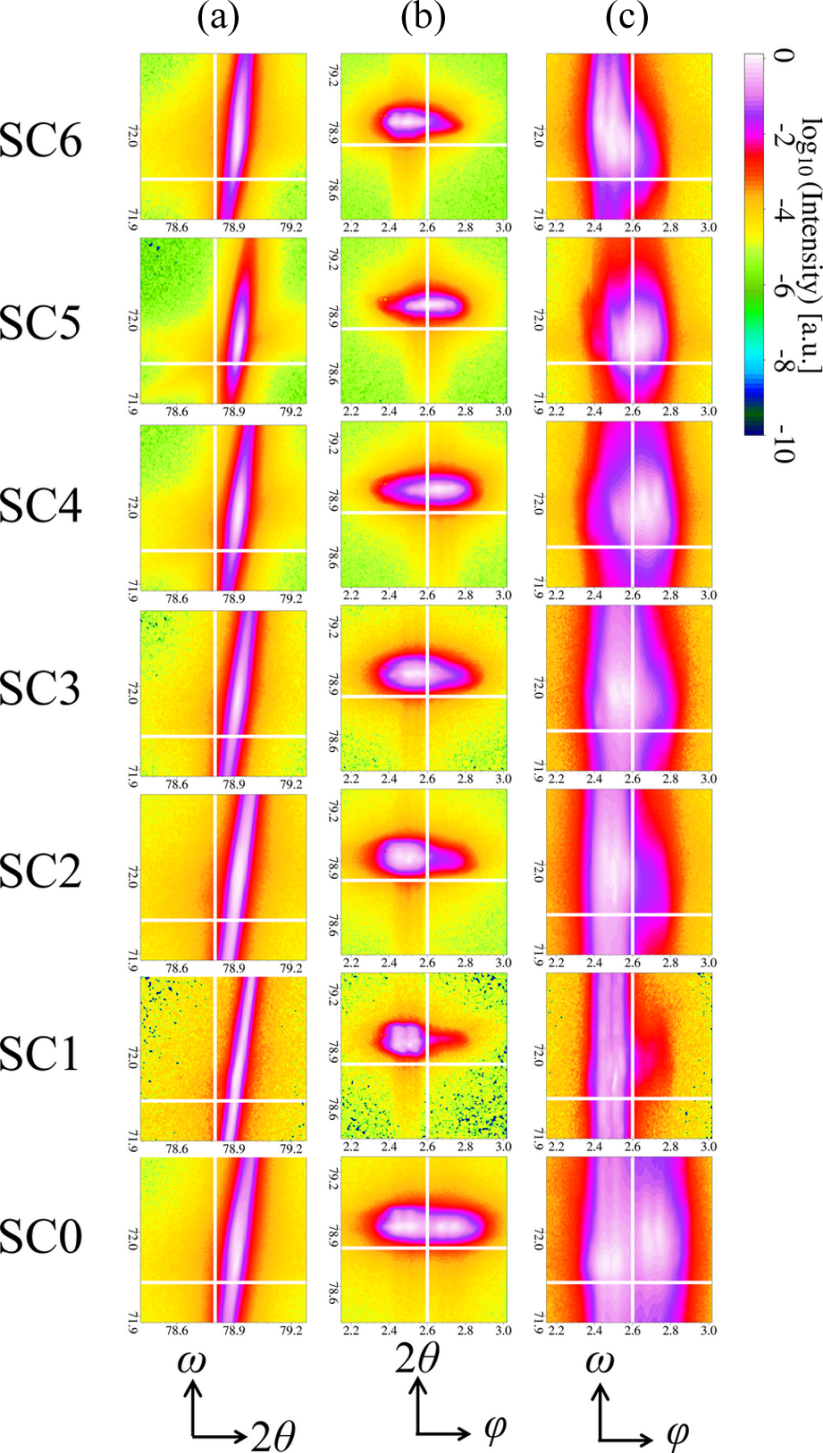
Matrix of averaged 2D 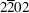 intensity profiles from the cluster 7 area. Each profile is the averaged map of all sampling points from the same SC. The coloring of the 2D profiles is based on logarithmic intensity. (*a*)–(*c*) Columns of raw 3D diffraction patterns projected onto 2D space. Columns (*a*), (*b*) and (*c*) in the matrix are for ω–2θ, 2θ–φ and ω–φ spaces, respectively. Each row of the profiles is labeled according to the UMAP cluster map in Fig. 12. For each image, a white crosshair is used as a guideline to help determine the positions of the diffraction peaks. Because of the zone plate in the optical system of nanoXRD, two separate peaks are observed along the φ direction. The values of φ are relative since a reference diffraction point along the φ direction is not given.

To give a further comparison of the differences between SCs, we averaged the 2D 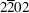 intensity profiles of all sampling points from the same SC and show the results in Fig. 13 ▸. To summarize briefly, all peaks except for those of SC5 exhibit similar large broadening along the ω direction, while there are no significant shifts in either the ω or 2θ directions. Compared with the ω and 2θ directions, the variation in the peak intensity profile in the φ direction is more pronounced with each different SC. On the basis on the characteristics of this φ variation, we can categorize the SCs into the following three types: (1) peaks are distributed on both sides of φ = 2.6° [white vertical lines in Figs. 13 ▸(*b*) and 13 ▸(*c*)] for SC0 and SC5; (2) peaks are primarily distributed on the smalle-angle side for SC1, SC2, SC3 and SC6; (3) peaks are distributed mainly on the larger--angle side for SC4.

Firstly, we infer that SC0 and SC5 in the first type are formed from different origins. SC0 represents the stem structure because SC0 encompasses the richest data features that allow us to observe peaks distributed over a wide range of φ direction. In contrast, SC5 is located away from the stem, with a relatively sharp peak around the center representing a data structure with a relatively lower defect density. As shown in Fig. 12 ▸(*a*), most of the data in the second type are distributed on the lower side of the cluster 7 area with significant broadening in the ω direction, corresponding to a relatively lower crystallinity. However, as shown in Fig. 12 ▸(*b*), SC2 and SC3 are the branches that separate from the stem, indicating that their crystal structure shares the same origin as SC0. Besides, SC1 and SC6 are somewhat separated from the stem in Fig. 12 ▸(*b*), so these regions are likely to be derived from a different source than the area of SC2, SC3 and the stem. When we further focus on SC4 in the third type, a correlated distribution of the stem SC0, SC4 and SC5 implies that SC4 represents the transition area of the crystal structure from a region with high defect density (SC0) to a region with low defect density (SC5). Interestingly, the considerable distance between SC6 and the other SCs in the UMAP plot means that SC6 has a weak relationship with the other SCs. However, contrary to such a large distance, the SC6 area connects directly with the SC5 area, as shown in Fig. 12 ▸(*a*). This structural relationship between SC5 and SC6 is interpretable by assuming the occurrence of overgrowth: overgrowth from SC4 and SC5 that covers the crystal from SC6 can explain the continuity between SC4 and SC5 and the cluster distance between SC5 and SC6, regardless of the spatial proximity.

From the above analysis, the changes in the crystal structure within the cluster 7 region can be summarized: SC0, SC1, SC2 and SC3 probably share a similar origin of crystal structure, distinct from SC6; meanwhile, the broadened diffraction patterns along the ω direction imply areas SC0, SC1, SC2, SC3 and SC6 have a relatively high density of defects, *i.e.* stacking faults and misfit dislocations. As the crystal grows, the defect density gradually decreases, leading to the formation of the SC4 region. The crystals from SC4 cover the lower part of the cluster 7 region, *i.e.* SC6 (possibly by an overgrowth), ultimately resulting in SC5 with a rather perfect crystal structure. Thus, an inspection of the continuity and distance of UMAP clusters can give insight into the hidden origins of crystallinity and crystal growth mechanisms that are not readily accessible by conventional analysis.

## 3. Discussion and conclusion

To ensure the reproducibility of the results listed in the main text and supplementary notes, parameters of used algorithms are listed in Appendix *A*[App appa] and Appendix *B*[App appb]. As we described in earlier sections, UMAP can offer robust and efficient clustering and visualization of the complex nanoXRD raw data structure in low-dimensional space. Notably, the application of UMAP is closely linked to the hypothesis on the relationship between spectral nanoXRD data structure and crystal structure, *i.e.* that a continuously evolving crystal structure is reflected in a continuous data structure, as shown in Section 2.2. The results of simulated 1D XRD data sets support the claim that UMAP efficiently identifies points where the structural continuity is disrupted, providing insights into potential microstructural transformations.

We believe that UMAP has the potential to generalize to other nanoXRD data sets, as its core mechanism does not rely on data-set-specific assumptions. The present study demonstrates the general applicability of UMAP to nanoXRD data sets, given that the analyzed data sets exhibit several typical features found in nanoXRD measurements of cross-sectional bulk crystals. These include continuous variations in diffraction patterns associated with structural evolution, as well as complex nonlinear changes that disrupt structural continuity due to microstructure formation during crystal growth.

As shown in Supplementary Note 7, applying UMAP in combination with agglomerative hierarchical clustering outperforms *t*-SNE and other dimensionality reduction methods, particularly in balancing the interpretability and structural representation of the data. We further compare UMAP results with those obtained by clustering directly on the raw data in Supplementary Note 7. We conclude that, although clustering on raw data provides meaningful insights, its reduced sensitivity to local data differences and its inability to give a visual guide to the relationships between clusters significantly limit its practical application. We note that methods such as spectral embedding yield clustering results most similar to those of UMAP, highlighting the potential of alternative approaches for nanoXRD data analysis in future studies. The findings in Supplementary Note 7 reinforce our confidence in UMAP’s potential applicability to other nanoXRD data sets, suggesting that it can serve as a powerful tool for analyzing complex diffraction patterns across a broad range of materials or measurement conditions.

In conclusion, in this research, we have examined the crystal structure evolution of an HVPE GaN sample using a position-dependent nanoXRD method. To overcome the challenges associated with conventional analysis, we applied a novel ML technique, UMAP, for analyzing position-dependent 3D (or 5D) diffraction data from nanoXRD experiments. Unlike conventional refinement processes that rely heavily on fit quality and diffraction peak properties, UMAP circumvents uncertainties by directly detecting and visualizing enormous numbers of XRD data structures from raw diffraction patterns taken at points.

We have evaluated UMAP’s performance in analyzing nanoXRD data sets from a cross-sectional HVPE GaN wafer, interpreting its results based on 2D projections of the raw 3D nanoXRD patterns. UMAP’s clustering capabilities have been explored, revealing the similarity and continuity of the diffraction pattern data. Notably, we observed significant differences between 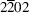 and 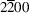 diffraction results for identical regions. This distinction is attributed to the relationship between data structure and recorded crystallographic information in the XRD patterns; the 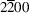 diffraction pattern reflects changes in *m* planes, while the 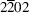 diffraction pattern primarily conveys *c*-plane characteristics. We further examined representative structural changes of the sample based on the characteristics of clusters obtained through UMAP. Specifically, we inferred possible defects present in the GaN crystal and their introduction mechanisms, as well as the modes and mechanisms of crystal growth.

Our findings demonstrate that UMAP effectively distinguishes between complex diffraction patterns while simultaneously enabling visualization and clustering for the exploratory analysis of nanoXRD data sets. Unlike conventional methods, UMAP makes use of all features of the data rather than relying solely on peak-related information. Clusters produced by UMAP allow for deeper insight into the crystal structure based on the diffraction data, enhancing the review of similarities and continuities between patterns. The proposed method shows promise for broader spectroscopic or diffraction-based analyses, such as Raman spectroscopy and scanning/tunneling electron microscopy diffraction imaging (4D-STEM). These results highlight UMAP’s versatility and its potential to improve analysis and experimental efficiency across various physics and materials science applications.

## 4. Related literature

For further literature related to the supporting information, see Berger *et al.* (2010 ▸), Cullity & Stock (2001 ▸) and Hagberg *et al.* (2008 ▸).

## Supplementary Material

Further discussion and additional figures. DOI: 10.1107/S1600576725004169/nb5399sup1.pdf

## Data Availability

Raw data were generated at SPring-8. Derived data supporting the findings of this study are available from the corresponding author Z. Wu upon reasonable request.

## Appendix A Hyperparameters of UMAP

UMAP is a flexible nonlinear dimensional reduction algorithm sensitive to the set of hyperparameters and randomness introduced to the algorithm. In this research, we set the primary hyperparameters as

n_neighbors=4,

min_dist=0.001,

n_components=2 and

metric=’euclidean’

and checked that the produced results and their shapes are robust and reproducible against the randomness.

## Appendix B Parameters of clustering and alternative dimensionality reduction methods

Regarding the settings and parameters used in the agglomerative hierarchical clustering method, the clustering was performed using the *scikit-learn* (Version 1.2.2) library in Python (Pedregosa *et al.*, 2011 ▸). The parameters were set as follows:

n_clusters=8

metric=’euclidean’

memory=None

connectivity=None

compute_full_tree=’auto’

linkage=’single’

distance_threshold=None

compute_distances=False

We adopted linkage=’single’ in clustering the UMAP embeddings, because our data exhibited clearly separated clusters alongside a prominent chain-like data structure. Single linkage is especially suitable for capturing such elongated or chain-like patterns effectively, making it the ideal choice for our UMAP analysis.

However, in scenarios involving raw data or other embedding plots, we strongly recommend the ’ward’ linkage due to its robustness against noise and outliers, ensuring more stable and balanced clusters in cases where clear separations were less evident.

For the visualizations presented in the main text, the number of clusters was selected subjectively based on objective calculations to ensure clarity and interpretability. At this stage, clustering primarily serves as a tool to visualize the relationship between data structure and crystal structure, rather than as an independent analytical objective. The specific cluster assignments are not of primary importance, as they are used to explore the data structure of the patterns rather than to establish definitive classifications. More discussions of the choice of the number of clusters can be found in Supplementary Note 2.

We acknowledge that this selection process is not yet fully optimized. In future work, we aim to refine the criteria for determining the number of clusters to ensure that cluster assignments are both interpretable and reproducible, aligning more closely with the sample’s underlying physical and structural properties.

When comparing with other dimensionality reduction methods performed by the *scikit-learn* (Version 1.2.2) library in Python (Pedregosa *et al.*, 2011 ▸), we set the parameters as follows:

(i) Spectral clustering:

n_clusters=8

eigen_solver=None

random_state=42

n_init=10

gamma=1.0

affinity=’nearest_neighbors’

n_neighbors=10

eigen_tol=0.0

assign_labels=’kmeans’

degree=3

coef0=1

kernel_params=None

(ii) PCA:

n_components=2

copy=True

whiten=False

svd_solver=’auto’

tol=0.0

iterated_power=’auto’

n_oversamples=10

power_iteration_normalizer=’auto’

random_state= 42

(iii) t-SNE:

n_components=2

perplexity=4

early_exaggeration=12.0

learning_rate=’auto’

n_iter=1000

n_iter_without_progress=300

min_grad_norm=1e-07

metric=’euclidean’

metric_params=None

init=’pca’

verbose=0

random_state=42

method=’barnes_hut’

angle=0.5

n_jobs=-1

square_distances=’deprecated’

(iv) MDS:

n_components=2

metric=True

n_init=4

max_iter=300

verbose=0

eps=0.001

n_jobs=-1

random_state=42

dissimilarity=’euclidean’

normalized_stress=’warn’

(v) Isomap:

n_neighbors=4

radius=None

n_components=2

eigen_solver=’auto’

tol=0

max_iter=None

path_method=’auto’

neighbors_algorithm=’auto’

n_jobs=-1

metric=’minkowski’

p=2

metric_params=None

(vi) Spectral embedding:

n_components=2

affinity=’nearest_neighbors’

gamma=None

random_state=None

eigen_solver=None

eigen_tol=’auto’

n_neighbors=4

n_jobs=-1
