# Supplementary material for: Machine learning assisted nanobeam X-ray diffraction based analysis on hydride vapor-phase epitaxy GaN
Source: J Appl Crystallogr. 2025 Jul 8;58(Pt 4):1205–19. doi: 10.1107/S1600576725004169 (PMC12321036; doi:10.1107/S1600576725004169)
Supplement: Supplementary file 1 [file j-58-01205-sup1.pdf]

JOURNAL OF
APPLIED
CRYSTALLOGRAPHY

Volume 58 (2025)

Supporting information for article:

Machine learning assisted nanobeam X-ray diffraction based analysis on hydride vapor-phase epitaxy GaN

Zhendong Wu, Yusuke Hayashi, Tetsuya Tohei, Kazushi Sumitani, Yasuhiko Imai, Shigeru Kimura and Akira Sakai

Supporting information

S1. Supplementary Note 1:

Since UMAP is a stochastic algorithm, meaning that randomness is used to accelerate various computational steps, its stability can vary. Although the official documentation states that UMAP is relatively stable (**McInnes *et al.*, 2018, McInnes *et al.*, 2018**), we observed that its results can fluctuate significantly in certain cases, particularly when the continuity of the data structure is relatively weak. Therefore, when analyzing nanoXRD datasets in this work, it is crucial to validate the robustness of UMAP plots against randomness.

As shown in Figs. S1 and S2, for both $2\bar{2}02$ and $2\bar{2}00$ diffraction patterns, the resulting UMAP embeddings exhibit ideal consistency across different sets of random seeds.

Additionally, we assessed the robustness of UMAP embeddings against noise and randomness by calculating trustworthiness using *scikit-learn* (ver1.2.2) in Python (**Pedregosa *et al.*, 2011**). Trustworthiness quantifies how well the local structure of high-dimensional data is preserved in the low-dimensional embedding. To evaluate this, we introduce noise into the raw data by varying the signal-to-noise ratios (SNR), assuming a normal noise distribution ~ $N(0, \sigma^2)$, while ensuring that the modified data remained positive. As shown in Fig. S3, UMAP demonstrates stable performance against noise and randomness (trustworthiness close to the range 0.99 ~ 1) in terms of preserving local data structure.

S2. Supplementary Note 2:

We selected the number of clusters by objectively evaluating clustering validation metrics, specifically the Silhouette scores, while also subjectively ensuring clusters were visually distinct, intuitive, and practically interpretable. Our goal was to choose a cluster number that avoids overly fragmented clusters (indicating an excessive number of clusters) and excessively merged clusters (indicating too few clusters). The variation of Silhouette scores with the number of clusters is shown in Fig. S4, and we further visualized clustering results corresponding to the top five highest Silhouette scores.

We found that for both results of $2\bar{2}02$ and $2\bar{2}00$ diffractions, we can achieve an optimal balance between objective validation metrics and subjective interpretability.

In the case of $2\bar{2}02$ diffractions, selecting fewer than 8 clusters resulted in separate clusters merging into a single cluster. Conversely, selecting more than eight clusters caused unnecessary fragmentation of cluster C7 (Fig. 6(a)), splitting it into two clusters, which is beyond the need to separate the data with a different continuity. Thus, eight clusters were selected, which also corresponds to the highest Silhouette score.

For $2\bar{2}00$ diffractions, with a relatively large number selection of 8 clusters, we can avoid the unwanted merging of distinct clusters while maintaining a high Silhouette score, thereby ensuring both interpretability and quantitative robustness.

S3. Supplementary Note 3:

When we focused on the 1D XRD spectrum shown in Fig. 5(b) in the main text, we tried to calculate the peak and width of the spectrum with the conventional method. The Gaussian function is often used to fit the XRD spectrum in spite of its asymmetric shape. Due to the uncertain fitting and the lost information compared to the raw 3D diffraction patterns, the peak and width of the 1D XRD spectrum can only show vague crystal structure information of the investigated sample. We illustrate this by mapping the peak and width information in Fig. S5.

To evaluate the quality of the Gaussian fitting used in this work, we calculated the R-squared, root-mean-square deviation (RMSE), and reduced chi-squared values, as shown in Figs. S6 and S7. It is worth noting that while R-squared is typically used in linear regression to indicate how well the model explains variance in the data, a high R-squared value can still suggest that a non-linear model captures the overall trend. Meanwhile, RMSE directly quantifies the difference between observed and predicted values obtained through fitting. Chi-squared assesses how well the model fits the data while accounting for measurement uncertainties. A key factor in calculating chi-squared is the estimation of uncertainty for measured data points, which is assumed to follow a Gaussian distribution and is approximated by the standard deviation of the residuals between fitted and observed values.

Figures S6(a-3) and S7(a-3) indicate poor fitting for the ω-intensity profiles, whereas the fitting for the 2θ-intensity profiles is relatively accurate. Additionally, although the reduced chi-squared values for the φ-intensity fitting are relatively low, Figs. S6(c-1), S6(d-3), S7(c-1), and S7(d-3) reveal a poor match between the raw and fitted data due to the inherent presence of two peaks in the φ direction — these shortcomings of conventional Gaussian fitting highlight the necessity of applying machine learning methods for improved modeling.

S4. Supplementary Note 4:

We demonstrate that diffraction patterns are distributed based on their continuity within a cluster analyzed by UMAP. Figure S8 compares the distribution of varying diffraction patterns in the UMAP plot and corresponding measurement points for cluster D in Fig. 6(c) as a representative example. Although data are classified into the same cluster in the UMAP plot, three secondary clusters (SCs) continuously distributed in the UMAP plot are defined in Fig. S8(a). The distribution of the sampling

points in the UMAP plot coincides with the spatial positions (Fig. S8(b)), which is the expected behavior of UMAP. The crystal structure and resulting diffraction patterns continuously vary along the growth direction, which is reflected in continuous distributions in the UMAP plot.

Furthermore, we next sought to inform the difference between SCs (Fig. S8). Except for the common trend described in Fig. 9, e.g., diffraction peaks keep shifting along the φ direction, shrinking along ω direction, and almost staying still at the 2θ direction, some minor changes are observed. As shown in Fig. S9(a), across the boundary between SC 1 and SC 2, minor stripes marked by red arrows vanished; meanwhile, peaks shitf in the opposite ω direction. Similarly, during the transformation from SC 2 to SC 3, stripes marked by red arrows in Figs. S9(a) and (b) gradually shrink until disappearance, with the peak shift in the φ direction. It is concluded that when sampling points with a continuous trend of variation are reflected as continuous distributions in the UMAP plot, minor changes will further create some secondary clusters.

S5. Supplementary Note 5:

To show the effect of X-ray penetration on the $2\bar{2}00$ diffractions' results, we performed a brief comparison of diffraction patterns across boundaries of $2\bar{2}00$ and $2\bar{2}02$ diffractions, as shown in Fig. S10. Figure S10(b) and S10(c) compare two sets of parallel diffraction peaks distributed in the φ direction marked by 1, 1`, 2, 2` (see Fig. S10(b-5)) observed in the 2D diffraction patterns projected onto the ω-φ space. For the $2\bar{2}00$ diffractions, peaks 1 and 2 have a higher intensity at positions below Y = 17 µm (Fig. S10(b)), but their intensities gradually decrease as the sampling points approach the boundary around Y = 17 µm. Consequently, the intensity of peaks 1` and 2` increases and replaces peaks 1 and 2. Interestingly, we observed a similar phenomenon in the *c*-planes (Figs. S10(c)), where the replacement of peaks 1 and 2 by 1` and 2` occurs near the boundary Y = 27 µm.

To further demonstrate the change of peaks' intensity near boundaries quantitatively, Figs. S11 and S12 show the profiles of diffraction intensity along a specific ω value extracted from the 2D intensity maps projected onto the ω-φ space. The laterally drawn dashed lines in Figs. S11(b) and S12(b) mark the position of the ω for the Figs. S11(c) and S12(c), respectively. With the help of the 1D intensity profile, the phenomenon that peaks1 and 2 disappeared while peaks 1` and 2` become dominated is observed clearly in both $2\bar{2}00$ and $2\bar{2}02$ diffractions. It is worth noting that since peaks 1` and 1 are too close in Fig. S12, peak 1` is obscured by peak 1 before the boundary.

According to the supplementary Figures, the replacement of peaks 1 and 2 by 1' and 2' is observed clearly for both the *m*- and *c*-planes. On the other hand, to verify the effect of X-ray penetration, we conducted calculations relating to the penetration depth, and the X-ray propagation is illustrated in Fig. S13. The X-ray diffraction intensity decreases when propagating in the sample due to the

absorption of the sample. Therefore, we can determine the intensity I_x of the X-ray beam that exits the sample as a function of depth x **(Cullity & Stock, 2001)**,

$$I_x = I_0 \cdot \exp(-\mu x(1/\sin(\omega) + 1/\sin(2\theta-\omega)) \text{ — (S1)}$$

and μ represents the absorption coefficient, and we calculated the value equaling 330.5177 cm^{-1} with referring to the database XGAM. **(Berger *et al.,* 2010)** The penetration depth is defined as the depth when $I_x/I_0=1/e$, while the related lateral penetration length S shown in Fig. S13 is calculated and listed in the Table. S1.

We showed that when the diffracted beam intensity reduces to $1/e$ of the incident beam, $2\bar{2}00$ diffractions have a lateral penetration length of around 13 μm; this value is much longer than that of $2\bar{2}02$ diffractions, which is about 2 μm. The calculation of penetration depth means that the $2\bar{2}00$ diffraction can record the diffraction information originating from the sample at 13 μm ahead. This fact explains the reason why we observed that the replacement of peaks in the $2\bar{2}00$ diffractions occurs at about 10μm lower than the case of $2\bar{2}02$ diffractions.

S6. Supplementary Note 6:

As demonstrated in Supplementary Note 1, the robustness of UMAP plots against randomness must be verified. Figure S14 shows the results of C7 from $2\bar{2}02$ diffractions, which confirm the stability of the tree-like structure consisting of a stem and several branches (shape in (b) is also similar to (a) and (c) by considering a slight rotation). The results of automatic clustering on C7 using different methods are in Fig. S15. All clustering methods were implemented using the *scikit-learn* (ver1.2.2) library (**Pedregosa *et al.*, 2011**), except for the Louvain community detection method, which was imported from the *networkx* library (**Hagberg *et al.*, 2008**). For agglomerative hierarchy clustering, all parameters were kept at their default values except for *n_cluster* = 7, ensuring consistency with the number of clusters presented in the main text. For hierarchical density-based spatial clustering of applications with noise (HDBSCAN), all parameters were set to default except for *min_cluster_size* = 15. In the Louvain community detection method, we set *n_neighbors* = 4 to maintain consistency with the UMAP settings, while all other parameters remain their default values. Similarly, for spectral clustering, we set only *n_clusters* = 7.

Although clustering on the 2D UMAP plot produces reasonable results, as shown in Figs. S15(a) to (c), the clustering results obtained directly from the raw data, shown in Figs. S15(d) to (f), are difficult to interpret. Moreover, while the clusters in Figs. S15(a) to (c) provide insights for distinguishing the separated clusters and branches, the stem structure is also segmented. To better differentiate stems from branches, we manually classified them in the 2D UMAP plot, as described in the main text. While manual classification introduces a degree of subjectivity, it can be both useful and necessary

when dealing with complex structures that automated clustering methods struggle to capture. In this case, manual labeling likely provides a more intuitive segmentation, highlighting key relationships between data structure and crystal structure that might otherwise be overlooked. This is further validated by the average diffraction pattern shown in Fig. 13.

S7. Supplementary Note 7

We compared the clustering on raw data and embedded plots from various dimensionality reduction algorithms using agglomerative hierarchy clustering and spectral clustering.

Since the $2\bar{2}02$ diffraction patterns exhibit better continuity in overall data structure than the $2\bar{2}00$ diffraction patterns, we utilized the former for the comparison. We directly clustered raw data by agglomerative hierarchy clustering (Fig. S16(a)) and spectral clustering (Fig. S16 (b)); we further visualized the results by coloring the UMAP plot according to the clusters identified by each of these methods. When we attempt to obtain and cluster the embeddings from other dimensionality reduction algorithms, for consistency, we set *n_cluster* = 8 across all methods, and for t-SNE, we set *perplexity* = 4 to match the set of *n_neighbors* = 4 in UMAP. Parameters of agglomerative hierarchy clustering for the UMAP plots are listed in the Appendix. B. Parameters of agglomerative hierarchy clustering for other plots are: *n_clusters=2*, *metric='euclidean'*, *memory=None*, *connectivity=None*, *compute_full_tree='auto'*, *linkage='ward'*, *distance_threshold=None*, *compute_distances=False*, of which the parameter *linkage* is changed into '*ward*' due to the weak separation between clusters and potential effects of noise and outliers in other plots except for UMAP. All algorithms except for UMAP are imported through the *scikit-learn* (ver1.2.2) library while the rest of parameters can be found in the Appendix. B (**Pedregosa *et al.*, 2011**).

For the results obtained from the raw data, Figs. S16(a) and (b) show that the boundaries of clusters identified directly from the raw data do not completely align with those obtained through UMAP embeddings, especially at cluster boundaries, although a continuous data structure remains evident even in the raw-data clustering. These observations suggest that while direct clustering in high-dimensional raw data provides meaningful insights, it may be less sensitive than UMAP to subtle or significant local differences among clusters. UMAP's dimensionality reduction appears to clearly reveal local cluster structures by mitigating the curse of dimensionality.

The plots in PCA, t-SNE, and MDS show a circular shape similar to that of UMAP (Figs. S16(c) to (h)). However, PCA likely oversimplifies the data structure, as it continuously distributes all patterns without clear separations, potentially masking meaningful distinctions. While MDS and t-SNE provide some indication of data structure variations, their clusters are more sparsely distributed

compared to the compact formations observed in UMAP, making it difficult to achieve convincing clustering results based solely on visualizations. Compared to these four methods, Isomap and Spectral Embedding produce projections that deviate more significantly from UMAP and result in less interpretable data structures.

To further compare the clustering results of different methods, we mapped the clusters obtained from various methods (Fig. S17) and calculated the Adjusted Mutual Information (AMI) to measure the similarity between various results (Fig. S18). Fig. S17 shows a layered structure was consistently detected for all methods, which results in relatively high AMI scores across all methods.

The results from t-SNE are particularly interesting. While Fig. S16(d) does not exhibit clear separations between clusters, Fig. S17(d) provides useful information, including the boundaries of C6, C0, and C2 in the upper part of the sample, which correspond closely to the boundaries of SC4, SC5, and SC6 in Fig. 12. Although areas from the upper and lower parts of the sample are classified into the same clusters C0 and C1, it is reasonable to assume that increasing the number of clusters might allow for better separation. However, Fig. S19, which presents results with *n_cluster* = 11, shows that the upper and lower sample regions remain within the same cluster. This limitation is likely attributed to t-SNE's inability to generate dense intra-cluster point distributions or sufficiently large inter-cluster distances, making it difficult to achieve clear visual separations of clusters observed in UMAP. Consequently, while t-SNE provides some structural insights, it does not offer the same level of clarity in visualized cluster separation as UMAP.

In conclusion, UMAP embeddings effectively generate clusters with sufficiently dense intra-cluster distances and large inter-cluster separations, facilitating intuitive visual identification and accurate cluster classification. Compared to direct clustering on raw data, UMAP exhibits better sensitivity and precision to local data differences and clearly visualizes the underlying data structure. Therefore, compared to alternative methods, UMAP presents an optimal balance between interpretability and structural representation of the data, making it particularly suitable for high dimensional structure data analysis like this research.

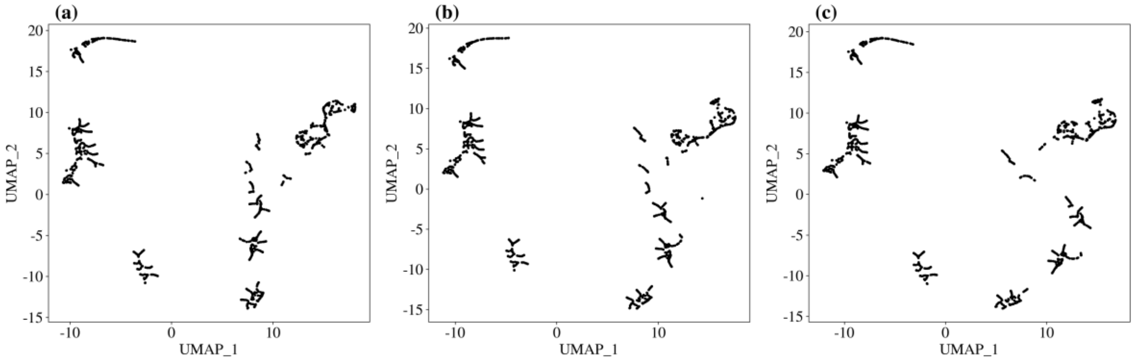

Figure S1 **UMAP plots for $2\bar{2}02$ diffraction patterns according to different sets of randomness.** (a) Random seed = 42. (b) Random seed = 123. (c) Random seed = 321.

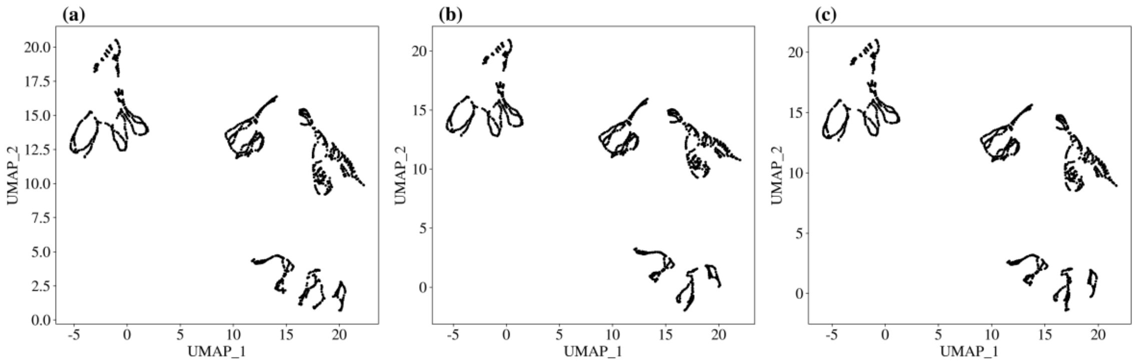

Figure S2 UMAP plots for $2\bar{2}00$ diffraction patterns according to different sets of randomness. (a) Random seed = 42. (b) Random seed = 123. (c) Random seed = 321.

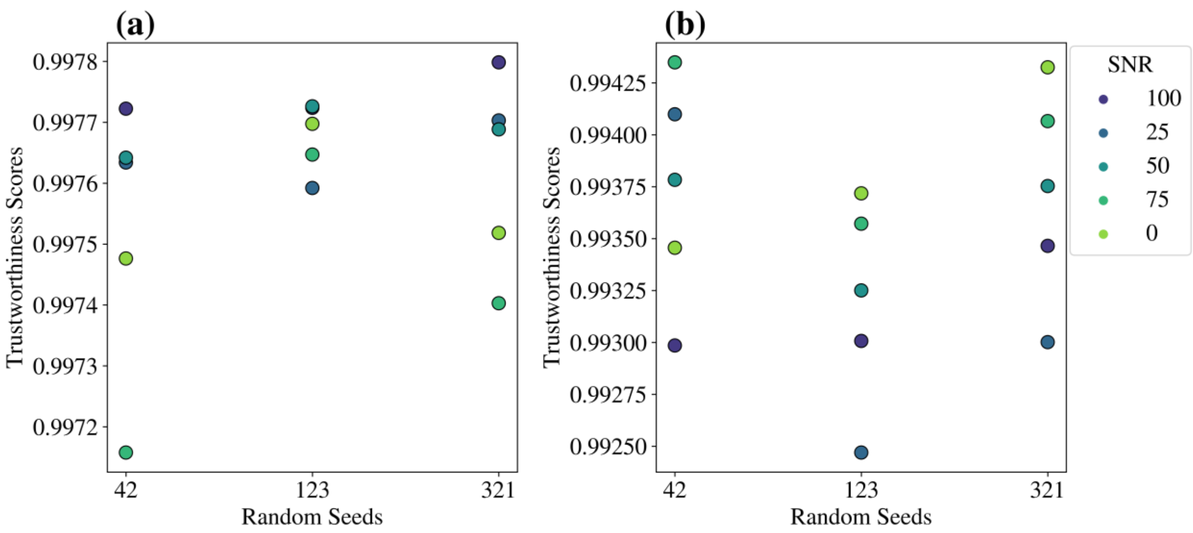

Figure S3 **Calculation results of trustworthiness against different random seeds and SNR.** (a) $2\bar{2}00$ diffraction. (b) $2\bar{2}02$ diffraction.

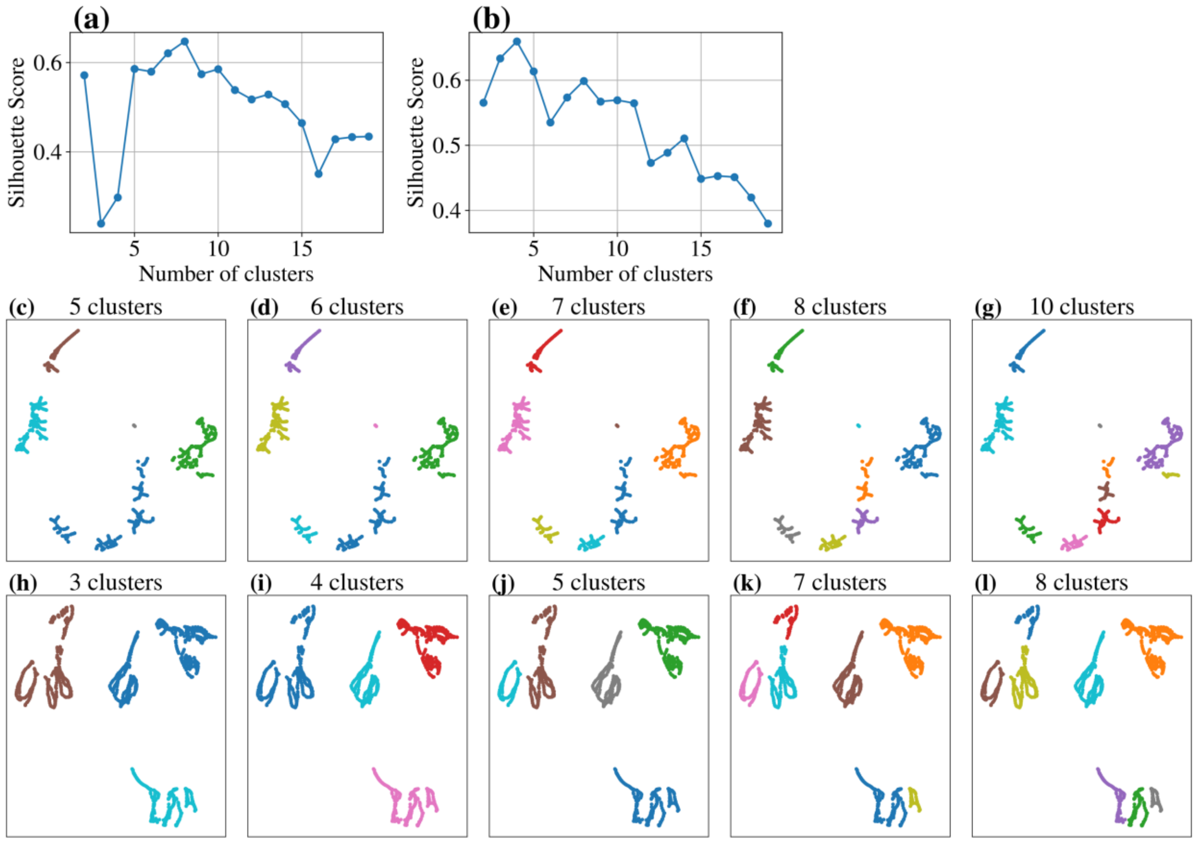

Figure S4 Effects of different numbers of clusters. The calculation of Silhouette scores vs. number of clusters for UMAP plots of (a) $2\bar{2}02$ and (b) $2\bar{2}00$ diffractions. To select the number of clusters with a proper balance between objective high scores and subjective interpretability, we plot the cluster results of the top 5 cluster numbers with the highest Silhouette scores. Results of $2\bar{2}02$ are listed in (c) to (g), while the results of $2\bar{2}00$ diffractions are listed in (h) to (l).

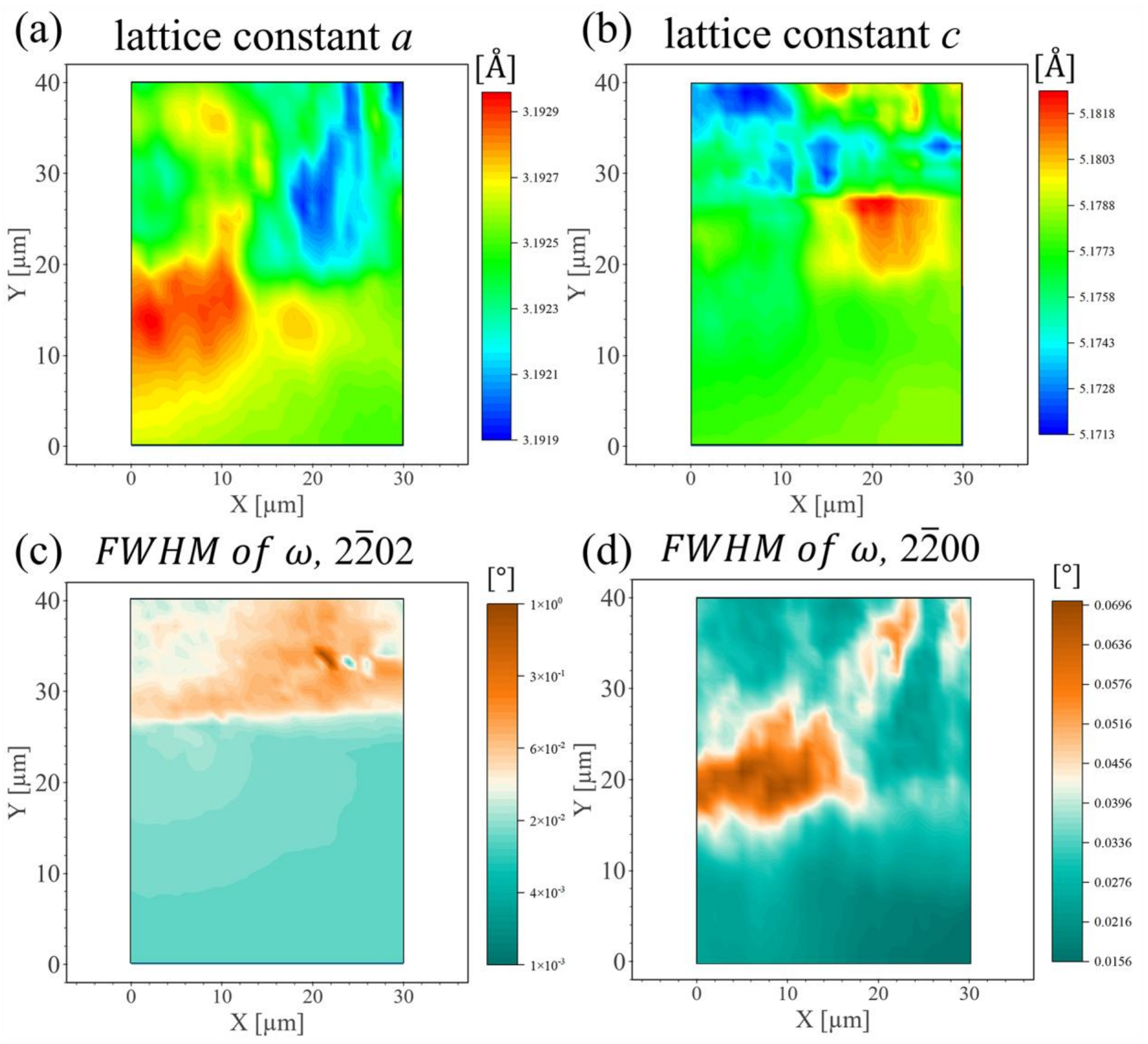

Figure S5 **Results obtained by the conventional method.** Lattice constant distribution maps of (a) *a* and (b) *c* calculated from peak values of 2θ. Lattice tilting fluctuation distribution maps of (c) $2\bar{2}02$ and (d) $2\bar{2}00$ diffractions are obtained from the full width at half maximum (FWHM) of ω. All maps are obtained from the 1D XRD spectra.

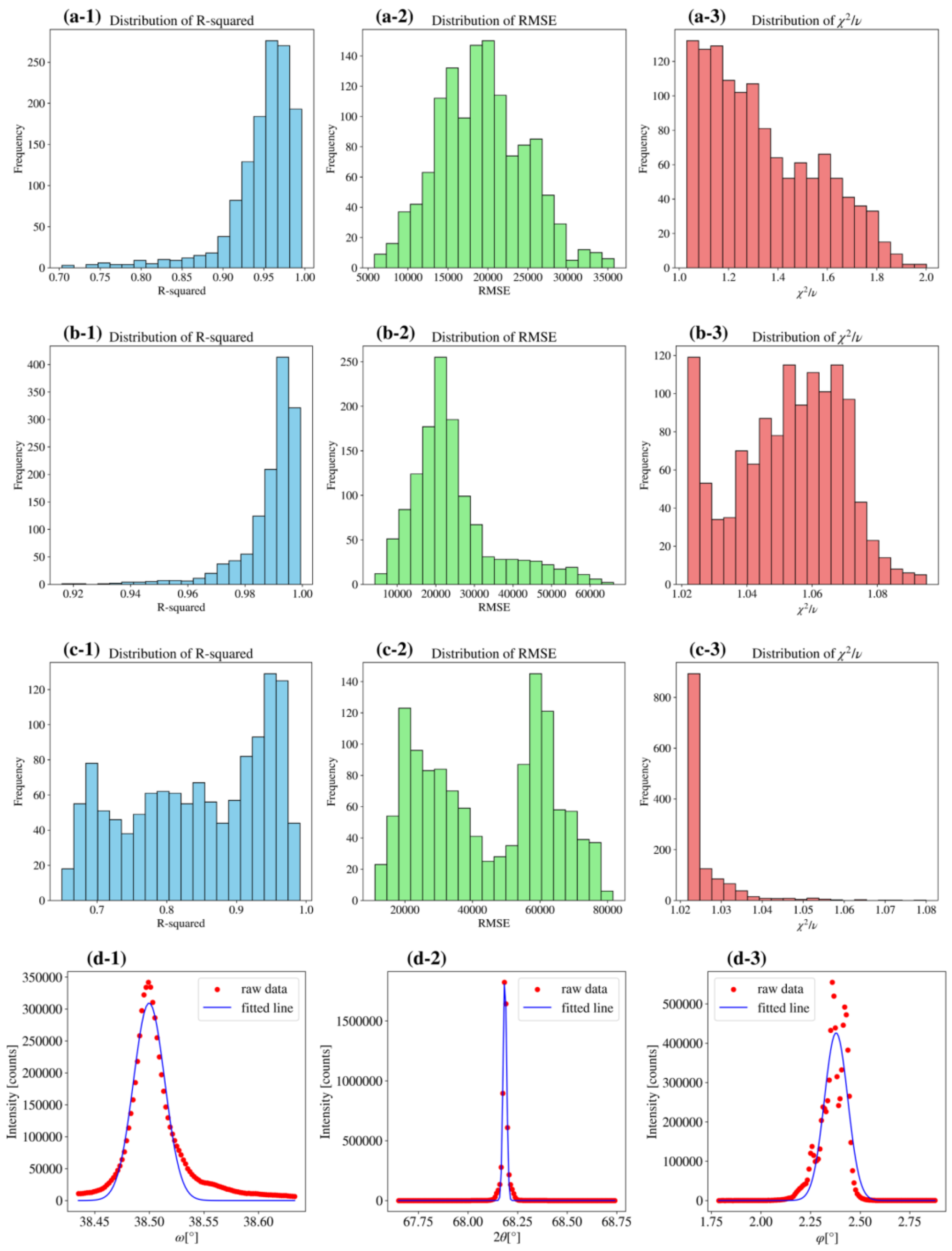

Figure S6 **Fitting quality of Gaussian fitting on 2$\bar{2}$00 diffraction results.** We list the fitting qualities of (a) ω-intensity, (b) 2θ-intensity, and (c) φ-intensity. From the left column to the right column in (a), (b), and (c), we plotted the distribution of R-squared, RMSE, and chi-squared, respectively. Representative comparisons between raw and fitted profiles are listed for (d-1) ω-intensity, (d-2) 2θ-intensity, and (d-3) φ-intensity.

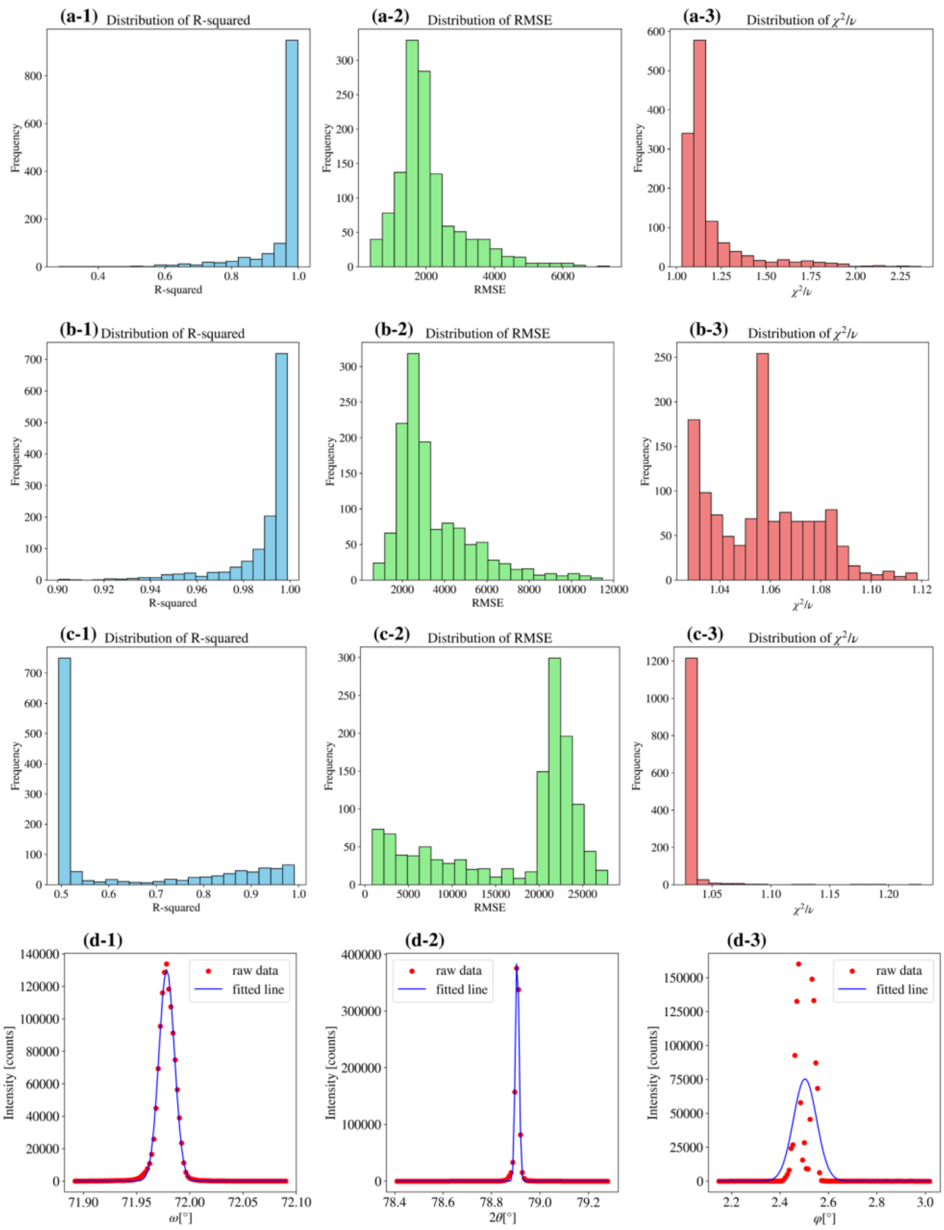

Figure S7 **Fitting quality of Gaussian fitting on 2$\bar{2}$02 diffraction results.** We list the fitting qualities of (a) ω-intensity, (b) 2θ-intensity, and (c) φ-intensity. From the left column to the right column in (a), (b), and (c), we plotted the distribution of R-squared, RMSE, and chi-squared, respectively. Representative comparisons between raw and fitted profiles are listed for (d-1) ω-intensity, (d-2) 2θ-intensity, and (d-3) φ-intensity.

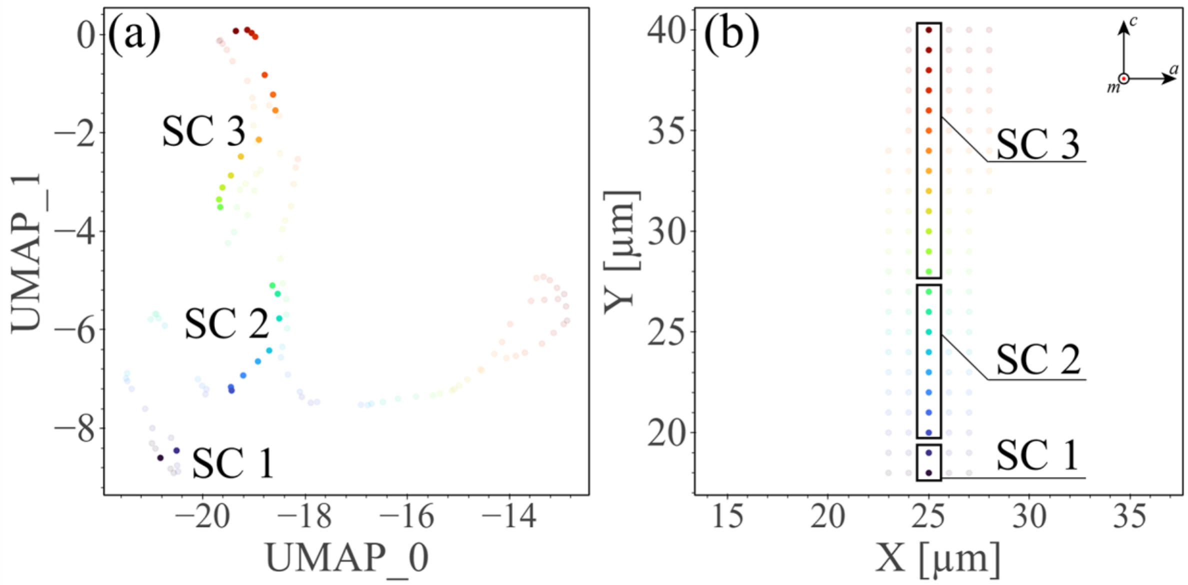

Figure S8 Comparison between the UMAP plot and corresponding distribution of measurement points. The results of (a) UMAP plot and (b) corresponding distribution of measurement points are from the cluster D in 2$\bar{2}$00 diffraction. Dark and light colors distinguish between the data from X = 25 μm and the rest. For the data of X = 25 μm, three secondary clusters (SCs) are marked. Points are colored based on the Y coordinates.

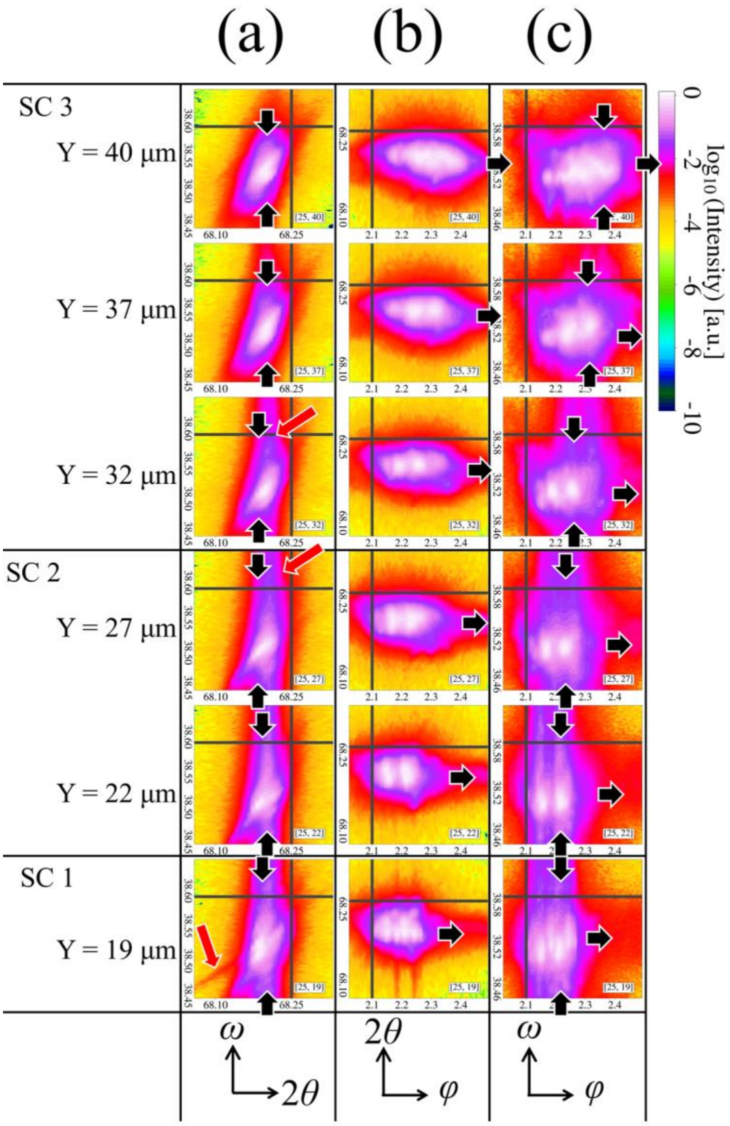

Figure S9 Comparison between UMAP clusters and representative 2D intensity profiles.

Results are from the cluster 3 of the $2\bar{2}00$ diffraction along the Y-direction in the sample. Each image is colored based on the logarithmic values of intensities. 2D intensity profiles of column (a) are ω-2θ results, those of (b) are the 2θ-φ results, while the ω-φ results are in column (c). We labeled each 2D intensity profile based on the secondary cluster in cluster 3 and used dark arrows to indicate the movement of the peaks' positions. Red arrows mark minor stripes near the peak.

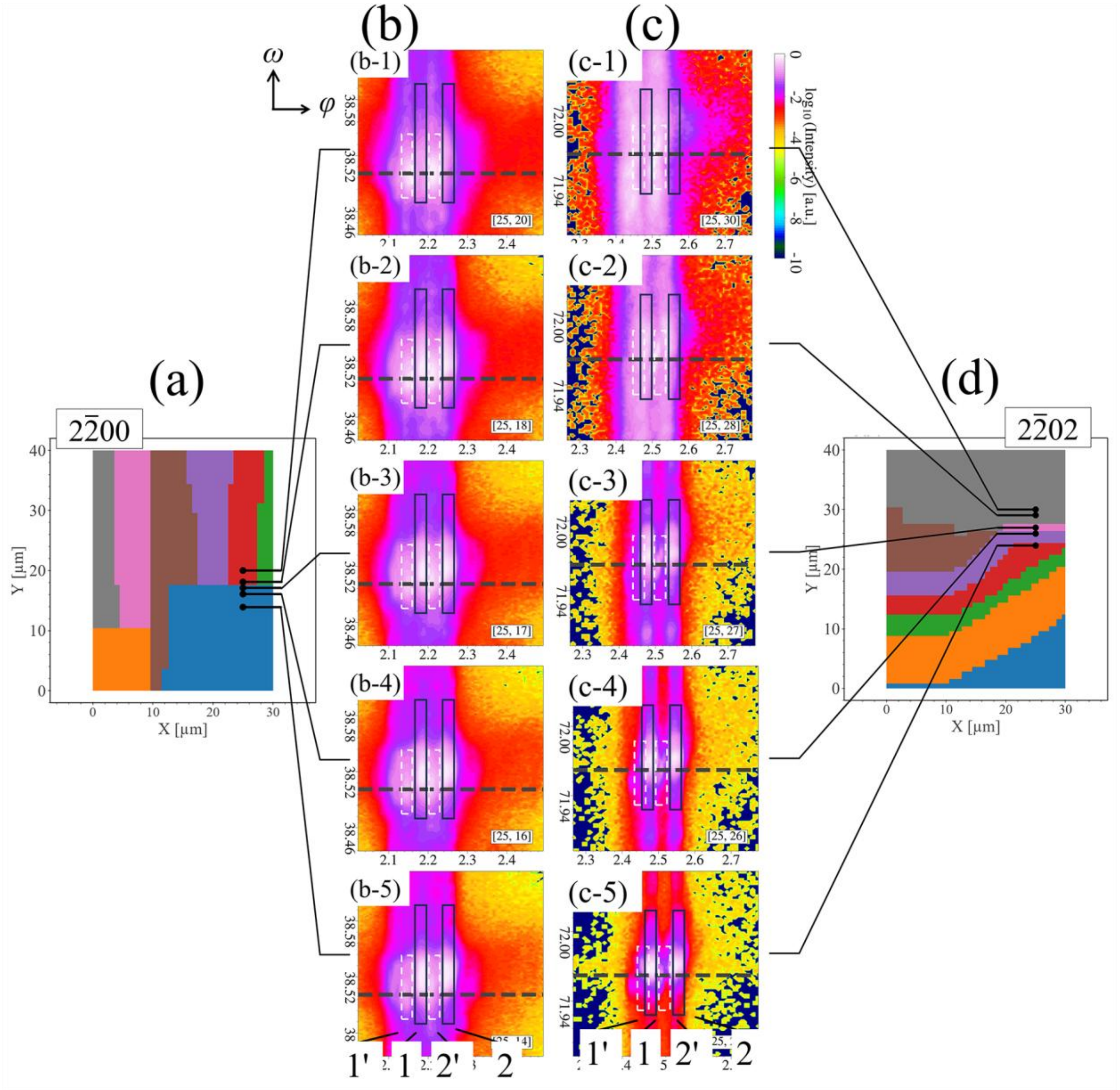

Figure S10 Comparison between representative 2D intensity profiles at ω-φ space. We imply the replacement of diffraction peaks parallelly distributed in the φ direction. (a) and (d) are UMAP cluster maps of $2\bar{2}00$ and $2\bar{2}02$ diffractions, respectively. (c) The corresponding 2D intensity profiles are from specific positions marked in (a), with parallel distribution of diffraction peaks 1, 1`, 2, 2`. (d) The corresponding 2D intensity profiles are from specific positions marked in (d).

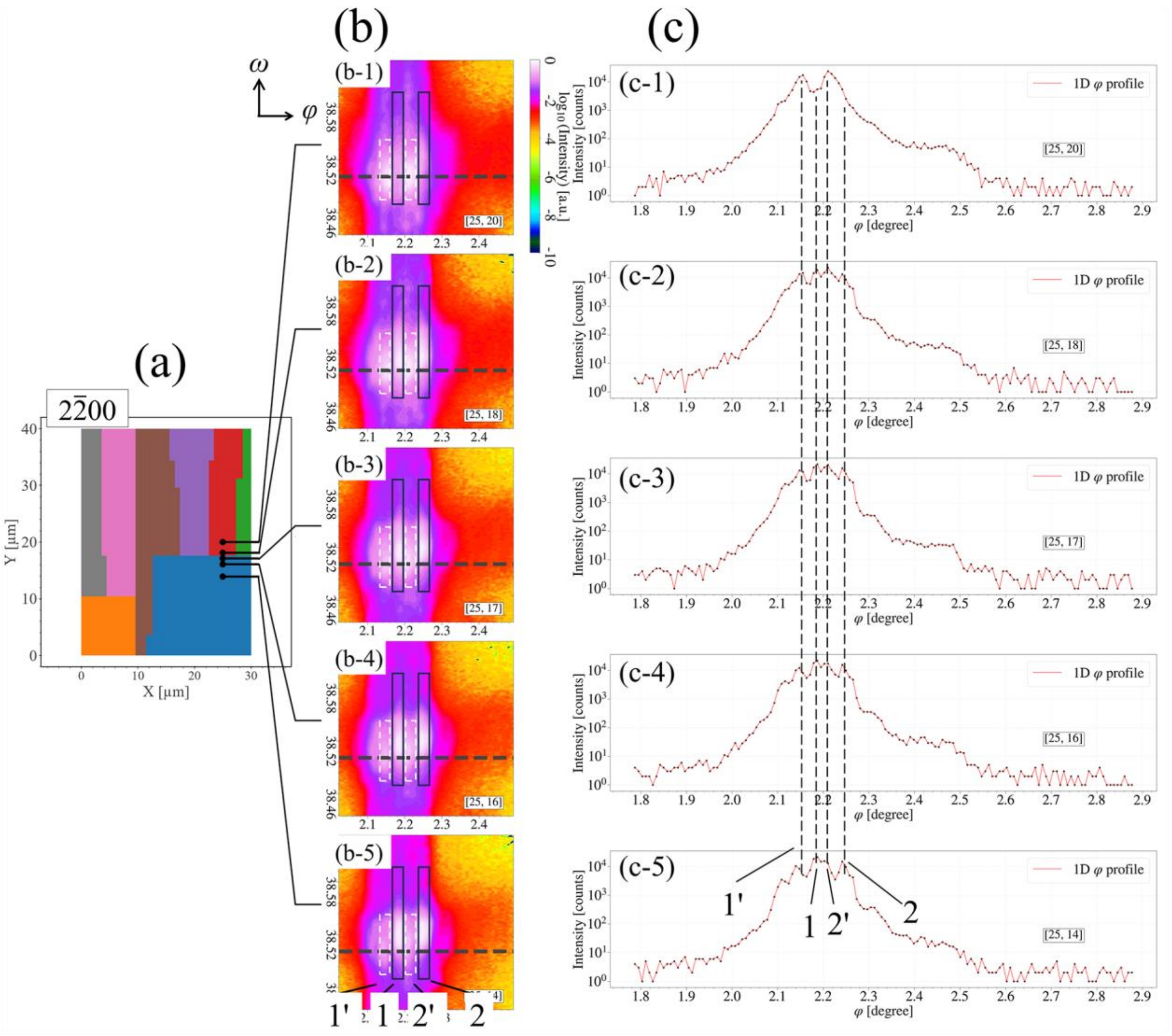

Figure S11 **Comparison between the UMAP cluster map and 2D intensity profiles from the 2$\bar{2}$00 diffraction.** The results of (a) UMAP cluster map, (b) 2D intensity profiles in the ω-φ space, and (c) corresponding distribution of diffraction intensity along ω = 38.5011° are obtained from the 2$\bar{2}$00 diffraction at positions close to the boundary Y = 17µm.

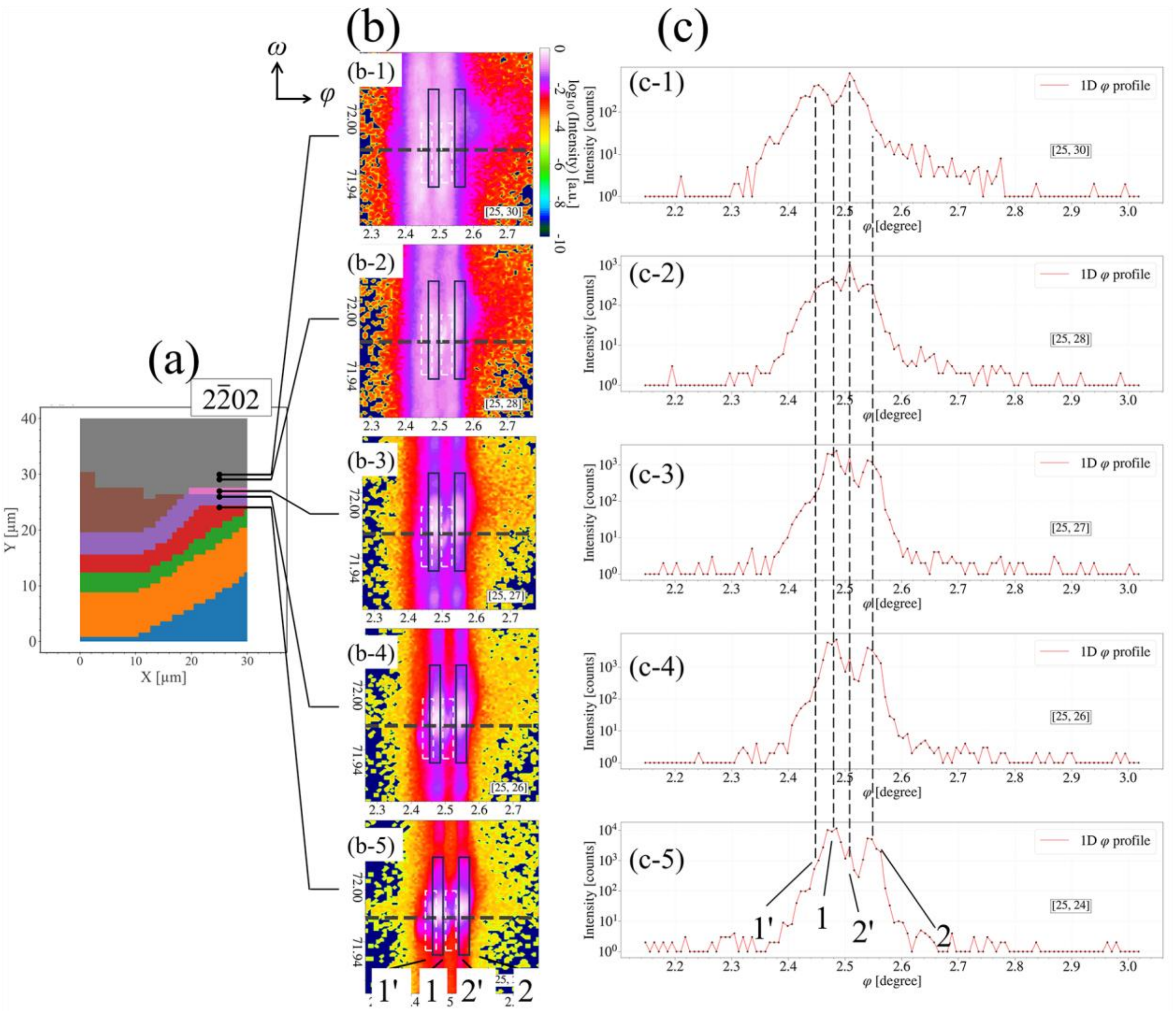

Figure S12 **Comparison between the UMAP cluster map and 2D intensity profiles from the 2$\bar{2}$02 diffraction.** The results of (a) UMAP cluster map, (b) 3D intensity projection on the ω-φ space, and (c) corresponding distribution of diffraction intensity along ω = 71.9622° are obtained from the 2$\bar{2}$02 diffraction at positions close to the boundary Y = 27 μm.

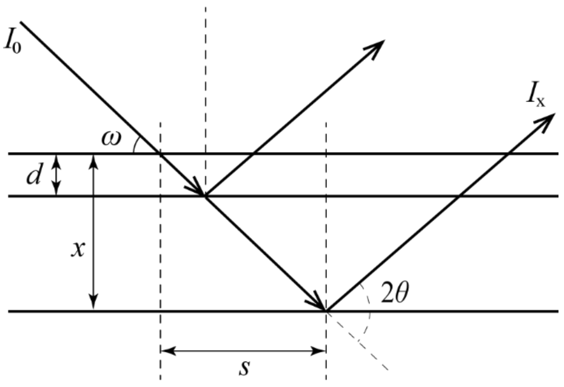

Figure S13 **Schematic of X-ray diffraction and penetration in the sample.** The diffraction occurred at every point along the propagation path after penetrating the sample, with intensity attenuation due to the mass absorption of the sample. I_0 is the intensity of the incident beam, and I_x refers to the intensity of the X-ray beam exits from the depth x.

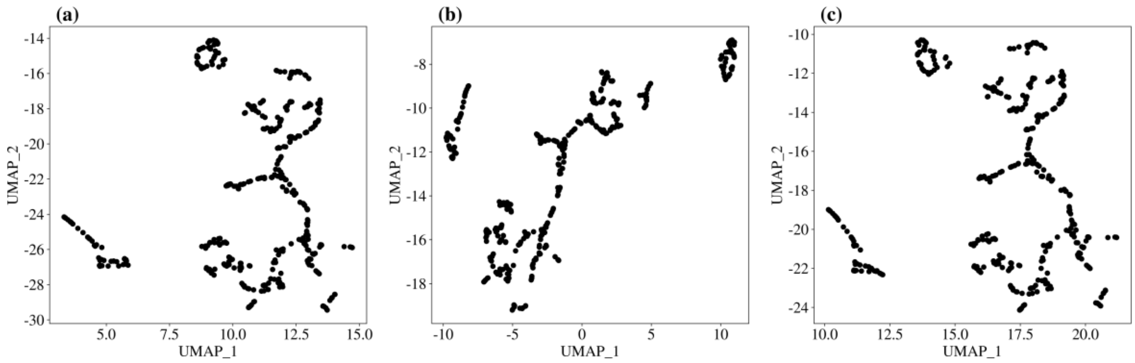

Figure S14 UMAP plots of C7 from 2$\bar{2}$02 diffractionss according to different sets of randomness. (a) Random seed = 42. (b) Random seed = 123. (c) Random seed = 321.

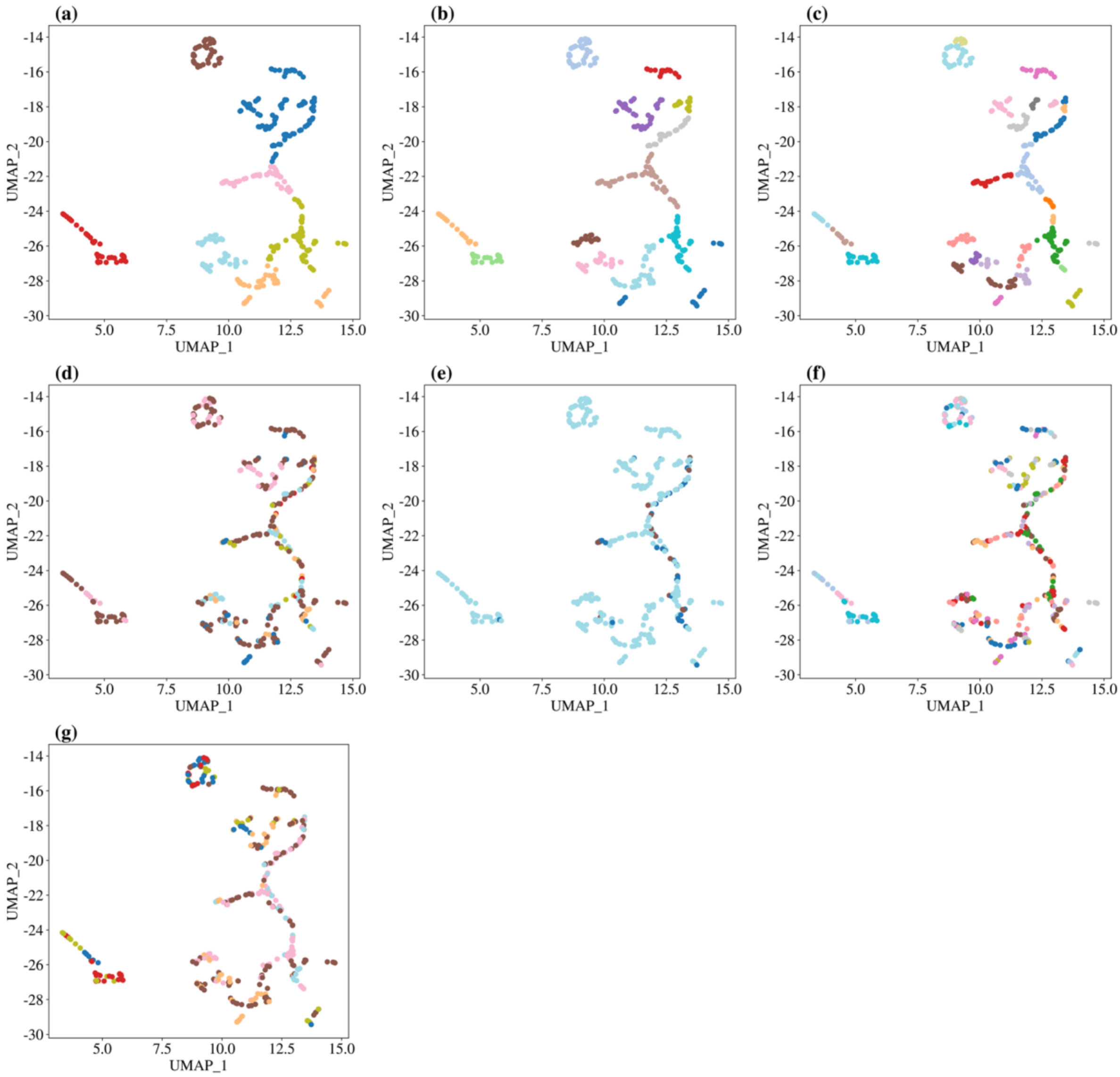

Figure S15 Representative clustering results according to different automatic clustering methods. The clustering results on the 2D UMAP plot are obtained from (a) The agglomerative hierarchical clustering method, (b) the HDBSCAN method, (c) the Louvain community detection method. We further listed the clustering results directly obtained from the raw data by (d) The agglomerative hierarchical clustering method, (e) the HDBSCAN method, (f) the Louvain community detection method, and (g) spectral clustering. Colors are based on clusters classified by each method.

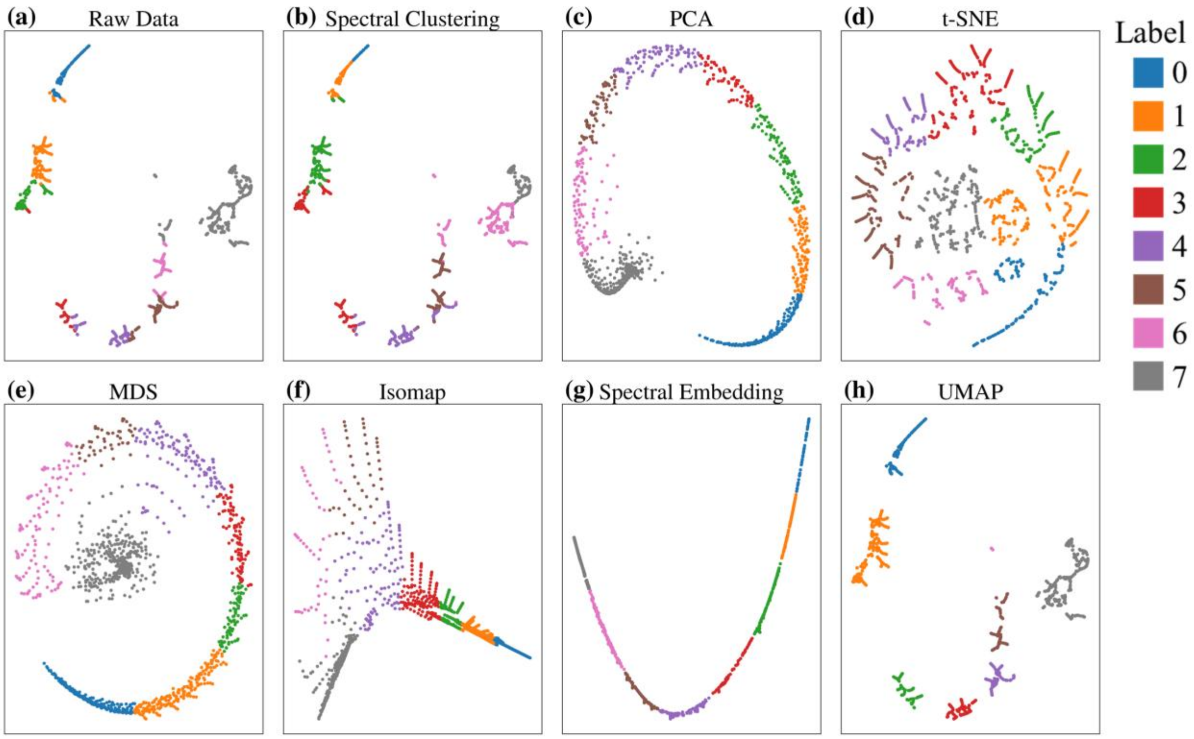

Figure S16 Comparison between embedded results from different dimensionality reduction methods. We compared the reduced $2\bar{2}02$ diffraction patterns from (a) raw data, (b) Spectral Clustering, (c) PCA, (d) t-SNE, (e) MDS, (f) Isomap, (g) Spectral Embedding, (h) UMAP.

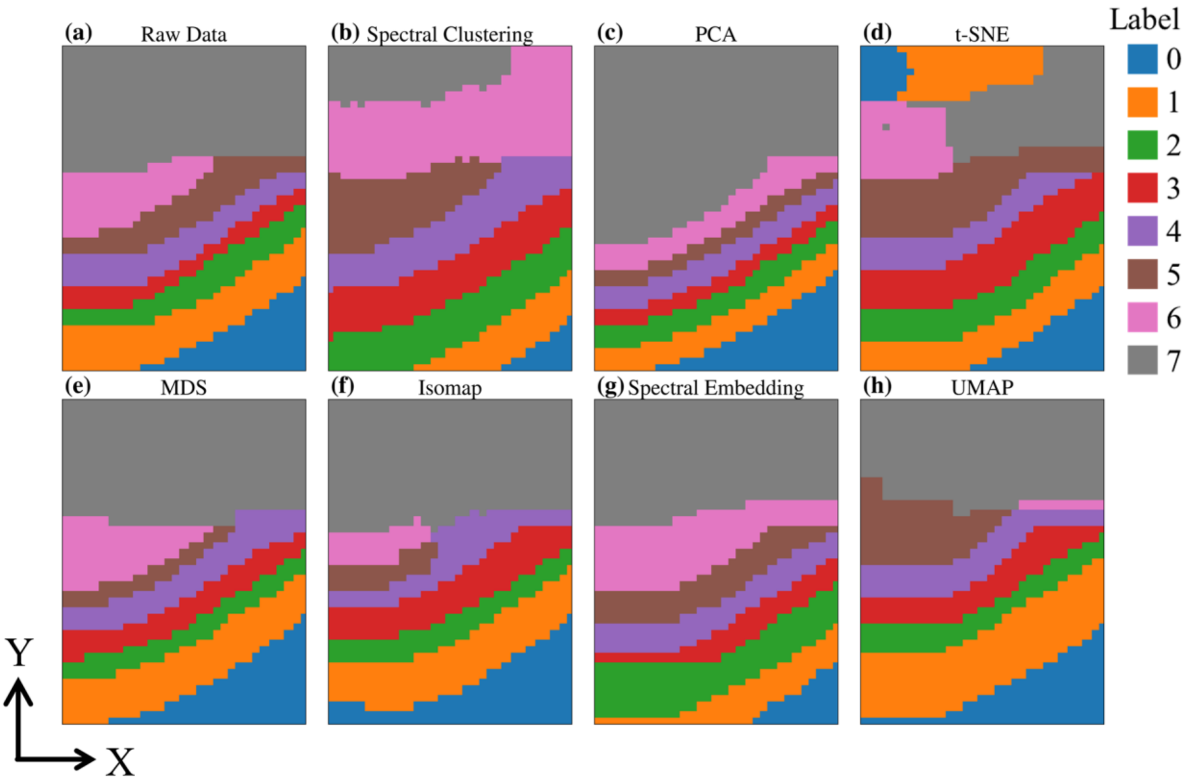

Figure S17 **Comparison between different clustering results on $2\bar{2}02$ diffraction patterns.** We compared and mapped the distribution of clustering results of $2\bar{2}02$ diffraction patterns by using (a) raw data, (b) Spectral Clustering, (c) PCA, (d) t-SNE, (e) MDS, (f) Isomap, (g) Spectral Embedding, (h) UMAP. For simplicity of comparison, the number of clusters in each method is set as eight. Except for the results in (b), clusters produced in other methods are classified by the agglomerative hierarchical clustering method, with the same set in the main text.

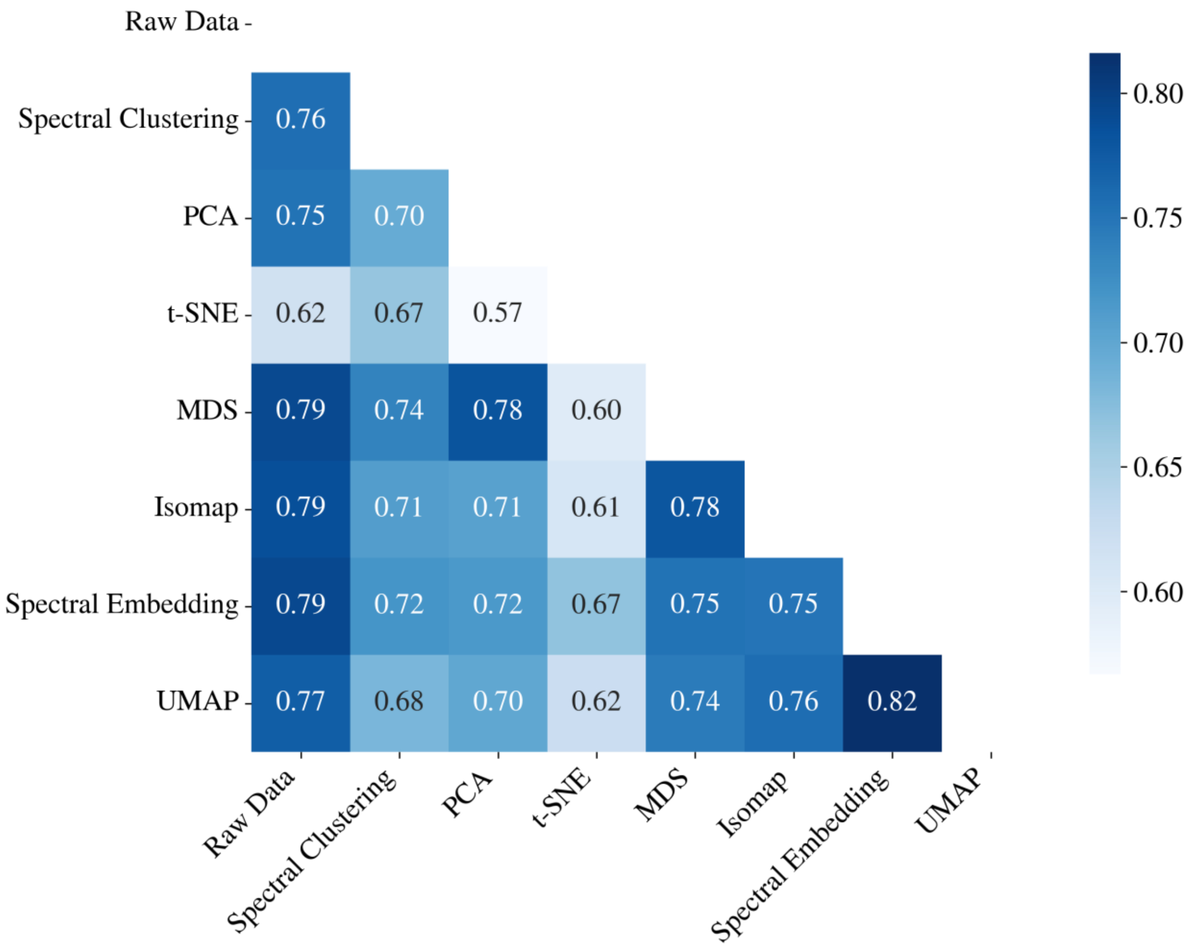

Figure S18 The Adjusted Mutual Information (AMI) scores between different clustering methods.

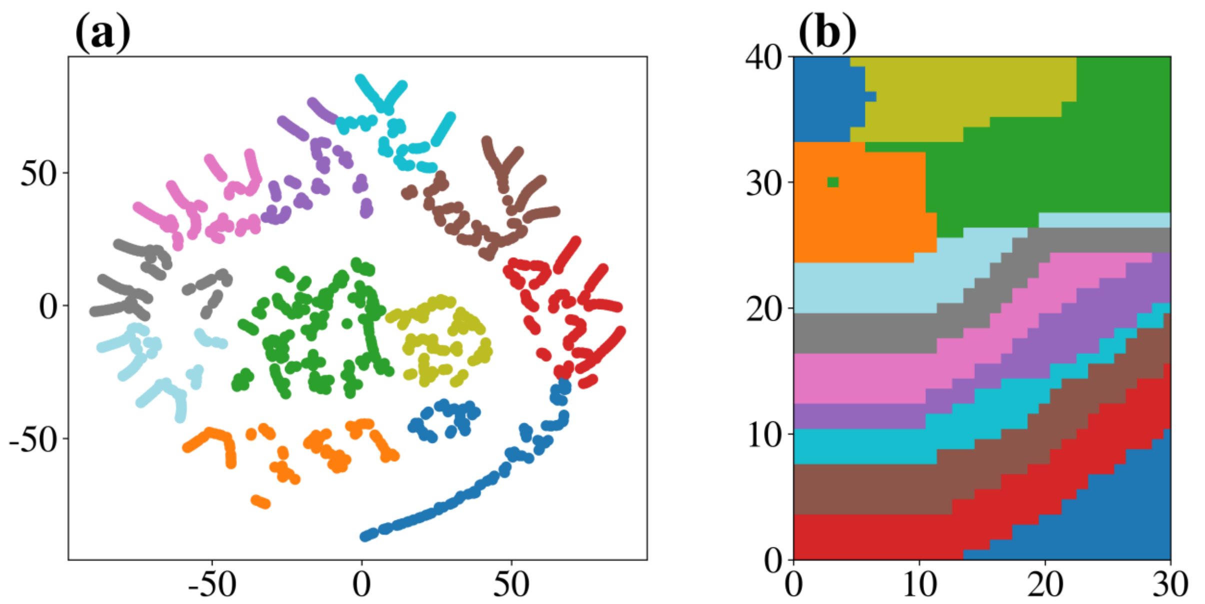

Figure S19 Clustering results by t-SNE on with a $2\bar{2}02$ diffraction patterns. (a) The clustering results from the t-SNE plot by the agglomerative hierarchical clustering method. Colors are based on classified clusters. (b) The corresponding distribution of clusters.

1 **Table S1** Lateral penetration length *S* of diffraction planes (Unit: μm)ss

Planes	*S* (μm)
**($2\bar{2}02$)**	2.411
**($2\bar{2}00$)**	13.227
